# Multimodal model integrating ultrasound and demographic data for the diagnosis of knee osteoarthritis

**DOI:** 10.1186/s12880-026-02249-8

**Published:** 2026-04-02

**Authors:** Takeharu Kiso, Yukinori Okada, Satoru Kawata, Kouta Shichiji, Eiichiro Okumura, Noritaka Hatsumi, Ryohei Matsuura, Masaki Kaminaga, Hikaru Kuwano, Erika Okumura, Narumi Nonoura

**Affiliations:** 1 Department of Radiology, Medical Corporation Seireikai Tachikawa Memorial Hospital, 2-12-14 Yakumo, Kasama, Ibaraki 309-1611 Japan; 2 Graduate School of Medical Sciences, Suzuka University, 1001-1, Kishioka- cho, Suzuka-shi, Mie, 510-0293 Japan; 3 Division of Radiation Oncology, Department of Clinical Medicine, Tokyo Medical University Hospital, 6-7-1 Nishi-Shinjuku, Shinjuku-ku, Tokyo, 160-0023 Japan; 4 Department of Radiology, Faculty of Medical and Health Sciences, Tsukuba International University, 6-20-1 Manabe, Tsuchiura-shi, Ibaraki 300-0051 Japan; 5 Postdoctoral Program, Graduate School of Health Sciences, Kyorin University, 5-4-1 Shimorenjaku, Mitaka-shi, Tokyo, 181-8612 Japan; 6 Department of Radiology, Tsukuba Medical Center Hospital, 1-3-1 Amakubo, Tsukuba City, Ibaraki 305-8558 Japan; 7 Department of Radiological Technology, Saitama Municipal Hospital, 2460 Mimuro, Saitama-shi, Saitama, 336-8522 Japan

**Keywords:** Deep learning, Knee joint, Osteoarthritis, Ultrasonography

## Abstract

**Background:**

Ultrasonography (US) is useful for soft tissue delineation, but its diagnostic objectivity is limited by surgeon dependency and lack of guidelines. Diagnostic support based on deep learning may overcome these issues, and integrating demographic factors (age, sex, and body mass index [BMI]) may improve accuracy. However, multimodal convolutional neural network (CNN) models incorporating these factors have not been fully investigated for knee osteoarthritis (OA). In this study, we developed such a model and evaluated its usefulness for diagnosis and severity classification.

**Methods:**

We developed image-only and multimodal CNN models using 11 architectures pre-trained on ImageNet. OA severity was classified based on Kellgren–Lawrence grades. Diagnostic performance was assessed using the area under the curve (AUC), sensitivity, specificity, positive predictive value (PPV), negative predictive value (NPV), and F1 score.

**Results:**

Among 491 limbs, 318 were diagnosed with OA. For diagnosing knee OA, a statistically significant improvement in performance of the multimodal model compared with the image-only model was observed only for AlexNet (AUC: 0.87 to 0.89, *p* = 0.036). No significant differences were observed between the models for other architectures, with differences in sensitivity, specificity, and F1 score remaining within 1–3%. SHapley Additive exPlanations analysis indicated that the contributions of age, sex, and BMI were generally small, and model predictions were predominantly driven by imaging features. In the stepwise evaluation, ResNet50 (image-only model) showed the highest sensitivity and NPV in the initial screening step (Step 1; 80% and 68%, respectively). For supplemental re-evaluation, ResNet152 and GoogLeNet demonstrated high specificity and PPV (96% and 97%, respectively). In severity classification among OA-positive cases (Step 2), VGG16 achieved the highest sensitivity and NPV (94% and 96%), whereas ResNet50 showed relatively high specificity and PPV (89% and 79%) for definitive diagnostic support following severity classification.

**Conclusions:**

CNN models using only US images demonstrated high diagnostic performance for knee OA. The addition of background factors provided limited benefit, suggesting that image-only models are sufficient for diagnostic support.

**Trial registration:**

This study was registered with the UMIN Clinical Trials Registration System: https://center6.umin.ac.jp/cgi-open-bin/ctr/ctr_view.cgi?recptno=R000056245; Trial Registration Number: UMIN000049395; Registration date: 2022/11/01.

**Supplementary Information:**

The online version contains supplementary material available at 10.1186/s12880-026-02249-8.

## Background

Osteoarthritis (OA) is a chronic joint disease that occurs frequently with aging and represents a major public health concern. Approximately 654 million people had knee OA globally in 2020, and this number is expected to increase with population aging [1]. OA is highly prevalent among older adults, with 60% of patients aged ≥ 55 years having radiographic evidence of OA [2]. It also places a significant economic burden on patients and reduces their quality of life owing to pain and limited range of motion [3, 4]. Furthermore, risk factors for OA are multifactorial, and obesity has been reported to be associated with a history of trauma and intense physical activity [5, 6].

The Kellgren–Lawrence (KL) classification based on radiographic images has been used to diagnose knee OA. However, it is based on bone structural changes, which limit the direct evaluation of soft tissue lesions, such as synovitis, joint effusion, and meniscus injuries [7, 8]. Therefore, magnetic resonance imaging (MRI) and ultrasonography (US) have been increasingly used in recent years [9–11]. MRI is excellent for detailed delineation of soft tissues. However, its high cost and long duration limit its use in routine practices [12]. In contrast, US is real-time, noninvasive, and economically superior in its evaluation. In particular, osteophytes depicted by US are the most widely accepted specific finding in the diagnosis of knee OA and have been incorporated into standardized scoring systems [13]. Conversely, joint effusions and synovitis are not specific findings in OA but are often observed in OA cases. These are ancillary indicators that reflect disease activity and progression, and their evaluation using US has been clinically useful [14].

Several US classification methods have been proposed for assessing the severity of knee OA: the grading scale by Mortada et al. [15] is a visual assessment based on the morphology of the medial femoral osteophyte, which is classified into five levels and reported to be in good agreement with the KL classification. In contrast, as a semi-quantitative method, Okano et al. [16] proposed a method to measure the size of osteophytes and classify them into four levels, and Yanagisawa et al. [17] reported a diagnostic method based on three criteria: osteophyte protrusion, joint cleft distance, and location of the outer margin of the meniscus. Kiso et al. [18] proposed the TOH-DBB index, a method that is less sensitive to knee alignment. Although all US evaluation methods have shown some success, the reliability of US-based diagnosis has been reported to be dependent on the examiner’s experience, skill, and subjectivity in interpreting findings, and its objectivity is limited [19]. Kiso et al. [20] conducted a radiomics analysis using features designed based on radiological knowledge and reported its diagnostic utility. However, radiomics analysis relies on handcrafted features that are predefined according to radiological expertise and optimized through statistical methods. Therefore, the feature space is inherently constrained, which may limit the ability to fully capture the high-dimensional and nonlinear information embedded within medical images.

Similar challenges exist in the traditionally used KL classification of X-ray images, both of which are based on visual evaluation; therefore, inter-observer variability is inevitable. To overcome these limitations and improve the objectivity and accuracy of diagnosis, image analysis based on deep learning has made rapid progress in recent years [21, 22]. High accuracy has been reported for KL classification of X-ray images using advanced convolutional neural networks (CNNs), such as ResNet, DenseNet, and EfficientNet [23, 24]. Furthermore, multimodal models integrating non-image data, such as age, sex, and body mass index (BMI), have been shown to improve diagnostic performance [25]. Pedoia et al. developed a DenseNet model integrating background factors (age, sex, BMI, and pain score) and T2 maps for the diagnosis of knee OA. This model achieved higher sensitivity (77.0%) and specificity (77.9%) than the model using only T2 maps (sensitivity 74.5% and specificity 76.1%). Similarly, Kim et al. [26] reported that integrating background factors with X-ray images improved the accuracy of the knee OA diagnosis model from 51.9% to 61.6%. They also introduced advanced architectures such as SE-ResNet and SE-ResNeXt, which incorporate an attentional mechanism [27], and a 3D CNN was developed for MRI [28]. However, the applications of deep learning targeting ultrasound images remain limited. Burlina et al. [29] applied AlexNet to the diagnosis of myositis and reported a sensitivity of 81.6%, specificity of 68.6%, and diagnostic accuracy of 76.2% in the deltoid, biceps, and rectus femoris muscles. Sasaki et al. [30] also used VGG16 to detect osteochondritis dissecans of the elbow joint and reported an area under the curve (AUC) of 0.95, sensitivity of 94.0%, F1 score of 88.5%, and diagnostic accuracy of 89.2%. Although these findings demonstrate the potential of AI applications in US, only a few CNN studies have been conducted on knee OA.

This study is characterized by the use of an end-to-end deep learning framework based on CNNs, in which feature representations are automatically learned from raw images. Rather than relying on manually engineered features as in conventional approaches, the model autonomously acquires diagnostically meaningful hierarchical features.

The primary objective of this study was to determine whether US images can be input into a CNN model with sufficient accuracy for knee OA diagnosis. The second objective was to investigate whether the multimodal model improves diagnostic accuracy by integrating known risk factors for knee OA, such as age, sex, and BMI, with US images. This exploratory study aimed to compare the usefulness of US images alone with that of a multimodal approach incorporating background factors. The results can provide a basis for expanding the clinical applicability of US-based AI diagnostic models. Furthermore, in addition to improving the accuracy of knee OA diagnosis, this approach may promote standardization and homogenization of US findings, which are easily influenced by subjective factors, and may contribute to its future application in clinical practice.

We hypothesized that CNN models trained on US images could achieve high diagnostic accuracy for knee OA and that integrating background factors, such as age, sex, and BMI, would further improve the diagnostic performance.

## Methods

### Study design

This prospective observational study was conducted at a single institution. Ultrasound images acquired as part of routine medical care during the study period were serially collected under an approved protocol and subsequently used secondarily for research purposes. The data used in this study were derived from the first author’s previous study, and no additional cases were added or excluded for this study [20]. We applied a different analytical approach using the same dataset in this study.

### Ethical considerations

Informed consent was obtained from all patients. This study was approved by the Ethical Review Committee of Tsukuba International University (No. R07-11) and conducted in accordance with the principles of the Declaration of Helsinki. It was registered in the UMIN Clinical Trials Registration System, and the Standards for Reporting of Diagnostic Accuracy guidelines provided by the EQUATOR Network were followed [31].

### Case selection

Patients with and without OA who visited the Department of Orthopaedic Surgery at Tachikawa Memorial Hospital for knee pain between December 2022 and April 2024 and underwent their first knee radiography and knee US during the study period were included. The following patients were excluded: (a) those who underwent supine knee radiography; (b) those who had undergone knee arthroplasty; (c) those who had undergone anterior cruciate ligament reconstruction; (d) those who did not agree to participate in the study; (e) those for whom data could not be collected owing to the absence of a US examiner; (f) those with external knee OA; and (g) those with secondary knee joint OA (such as rheumatoid arthritis, gout/pseudogout, crystal-induced arthritis, and idiopathic medial femoral condylar osteonecrosis). Remeasurements performed by the same examiner were also excluded.

### Criteria for knee OA diagnosis

The chief complaint, physical examination, and radiographic findings were integrated to diagnose knee OA according to the European League Against Rheumatism criteria [32]. The radiographic images were evaluated by three orthopedic surgeons (with 29, 15, and 8 years of experience) based on the KL classification [33, 34]. The KL classification is defined as follows: Grade 0: no abnormalities; Grade 1: suspicious joint crevice narrowing and minimal osteophyte formation; Grade 2: obvious osteophyte formation and possible joint crevice narrowing; Grade 3: moderate osteophyte formation, obvious joint crevice narrowing, and some degree of sclerosis or osteophyte formation; Grade 4: large osteophytes, marked joint crevice narrowing, strong stiffening, and obvious deformity.

Specifically, KL grades 0–1 were classified as non-OA, whereas KL grades 2–4 were classified as OA. KL grade 2 is characterized by definite osteophyte formation and is commonly considered a threshold for the diagnosis of OA; therefore, this classification was adopted in the present study. Furthermore, for severity-based analyses, KL grades 3–4 were defined as severe OA, while KL grade 2 was classified as mild OA. The diagnosis was reached by consensus among three physicians, all of whom were blinded to US imaging. In cases where the evaluations were not concordant, the final diagnosis was determined through consensus after discussion that incorporated both clinical and imaging findings. Because the final diagnosis was established by consensus, inter-rater agreement metrics such as Cohen’s κ or Fleiss’ κ were not calculated in this study. These diagnostic criteria and case classifications were implemented in a previous study by the first author [20].

### Standing knee radiographic and ultrasonographic examinations

#### Standing knee radiography

Standing knee radiography was performed under fluoroscopic guidance using a SONIALVISION G4 radiography system (D150BC-40 S; Shimadzu Corporation, Kyoto, Japan). The patients stood on the affected leg on a vertical fluoroscopic table with their footrest. The fluoroscopy settings were as follows: pulse mode, N; pulse rate, 7.5 fps; and initial tube voltage, 50 kV. The imaging parameters were as follows: tube voltage: 60 kV; current-time product: 12.5 mAs; imaging duration: 25 ms; source-to-image distance: 150 cm.

#### Standing knee ultrasonographic examination

Standing knee US was performed on the same day as the standing knee radiography. US was performed using an Aplio400 (TUS-A400/W1, Canon Medical Systems Inc., Tochigi, Japan) with a 12 MHz linear probe (PLT-1204AT). The imaging settings included the following: dynamic range, 65; mechanical index, 1.2; tissue harmonic imaging type, Diff 18 M; Precision, 5; ApliPure, 8; Tissue Specific Optimization, 4; Time Smooth, 4; and gamma, 5. The gain, diagnostic depth, and receiver focus were adjusted for every patient. Image acquisition was standardized based on protocols from previous studies [18]. Additional file 1 Figure S1 shows a representative illustration of the US probe position.

The long-axis image of the medial joint cleft was obtained during the ultrasonographic examination of the knee in the standing position. The longitudinal section was placed parallel to the medial collateral ligament of the knee. The medial femoral epicondyle was used as a landmark. This position was reproducible by palpating the medial epicondyle before the examination and positioning the proximal end of the probe, accordingly. Caution was exercised while adjusting the angle and position of the probe to ensure clear delineation of the medial collateral ligament.

### Ultrasonographers and their reliability

The US images of the standing knee joint were obtained by two sonographers certified by the Japanese Society of Ultrasonography with 17 and 10 years of ultrasonographic examination experience, respectively. The diagnostic accuracies of the US images obtained by these examiners were validated in a previous study by the first author by comparing their diagnoses with the clinical diagnoses of three orthopedic surgeons [18]. Examiner A had a clinical OA diagnostic accuracy of 86.0%, with sensitivity, specificity, positive predictive value (PPV), and negative predictive value (NPV) of 93.0%, 77.0%, 82.0%, and 86.0%, respectively. The corresponding values for examiner B were 89.0%, 87.0%, 92.0%, 93.0%, and 86.0%, respectively. A similar trend was observed for the severity classification of OA (Examiner A: diagnostic accuracy, 86.0%; sensitivity, 93.0%; specificity, 77.0%; PPV, 82.0%; and NPV, 91.0%; Examiner B: diagnostic accuracy, 89.0%; sensitivity, 87.0%; specificity, 92.0%; PPV, 93.0%; and NPV, 86.0%).

### Model building, training, evaluation, and statistical analysis methods

#### Data set and preprocessing

The data of clinically diagnosed OA and non-OA cases were included; OA presence and absence were labeled as 1 and 0, respectively, based on the diagnosis by an orthopedic surgeon. A separate classification model was developed based on the data of cases diagnosed with OA to assess severity. The KL classification, which is widely used in orthopedics, was used as the standard for defining severity. A binary classification was performed, with grades 2 and lower representing mild OA (0) and grades 3 and higher representing severe OA. The images were preprocessed using ImageJ (ver. 1.53k, National Institutes of Health, Bethesda, MD, USA). The size of the region of interest (ROI) was set to 550 × 550 pixels to ensure the inclusion of key anatomical structures relevant to the diagnosis of knee OA, such as the meniscus and osteophytes, in all cases. A predefined macro script (Macro550.ijm) was used to automatically crop the ROI from each ultrasound image. The upper edge of the ROI was positioned to always include the skin surface, and the depth direction was fixed from this point. The horizontal position was determined based on the center of the probe length at the time of image acquisition, as the joint region was captured at the center during scanning. All ROIs were adjusted to exclude imaging artifacts and annotation overlays. Images were cropped using ImageJ, saved in JPEG format, and resized to 227 × 227 pixels. They were converted from BGR to RGB color modes, and their pixel values were normalized to a range of 0–1. Background factors (age, sex, and BMI) were standardized using StandardScaler in pandas and scikit-learn. Cases with missing data were excluded from the analysis. The background variables used in this study (age, sex, and BMI) were obtained from clinical records at the time of radiographic and ultrasonographic examinations. The data were split using stratified extraction. Data were partitioned by stratified sampling based on KL classification, with 70% for training and validation and 30% for testing. Data for training and validation were further split at a ratio of 8:2. When data of both knees from the same patient were available, each knee was treated as an independent sample, and dataset partitioning was performed at the knee level. To ensure reproducibility, the random seed was fixed (random seed = 42) for all processes, including data splitting and model training. The dataset was divided into training, validation, and test sets while maintaining comparable distributions of OA status and KL grades across the subsets. The numbers of cases, OA status, and the distribution of KL grades for each dataset are summarized in Table 1.

**Table 1:** Distribution of cases, OA status, and Kellgren–Lawrence grades across the training, validation, and test datasets

Dataset	*N*	Clinical OA (*n*, %)	Non-OA (*n*, %)	KL 0	KL 1	KL 2	KL 3	KL 4
Train	274	177 (64.6%)	97 (35.4%)	11	86	112	40	25
Validation	69	45 (65.2%)	24 (34.8%)	5	19	29	7	9
Test	148	96 (64.9%)	52 (35.1%)	9	43	60	22	14

KL, Kellgren–Lawrence; OA, osteoarthritis

#### Model development

Two types of CNNs were developed: an image-only model that used only US images as input and a multimodal model that integrated background factors (age, sex, and BMI) with image information. CNN architectures pre-trained on ImageNet were used as base models. In this study, all CNN backbones were initialized with weights pretrained on ImageNet, and end-to-end fine-tuning was performed without freezing any convolutional layers. They were DenseNet (169, 201), InceptionV3, Inception-ResNet V2, ResNet (50, 101, 152), Xception, VGG16, GoogLeNet, and AlexNet. These were adopted from previous studies [29, 30, 35–38] that reported high diagnostic accuracy. For example, a study using VGG16 reported an AUC of 0.95 and a sensitivity of 94.0% [30]. The diagnostic accuracies of the CNN models used in the aforementioned studies are presented in Additional file 2 Table S1. In the multimodal model proposed in this study, image features and background variables (age, sex, and body mass index) were integrated using a gated fusion mechanism. Specifically, the fusion process was defined as follows:

$$\:g=\sigma\:({W}_{g}y+{b}_{g}),{x}^{{\prime\:}}=x\odot\:g,f=[{x}^{{\prime\:}};y],$$

where $\:x$ represents the image features extracted by the convolutional neural network, $\:y$ denotes the background variables, $\:\sigma\:$ is the sigmoid activation function, and $\:\odot\:$ indicates element-wise multiplication. Through this mechanism, the multimodal model dynamically adjusts the contribution of image features according to the background information, thereby generating a fused feature representation $\:f$.

**Fig. 1:**
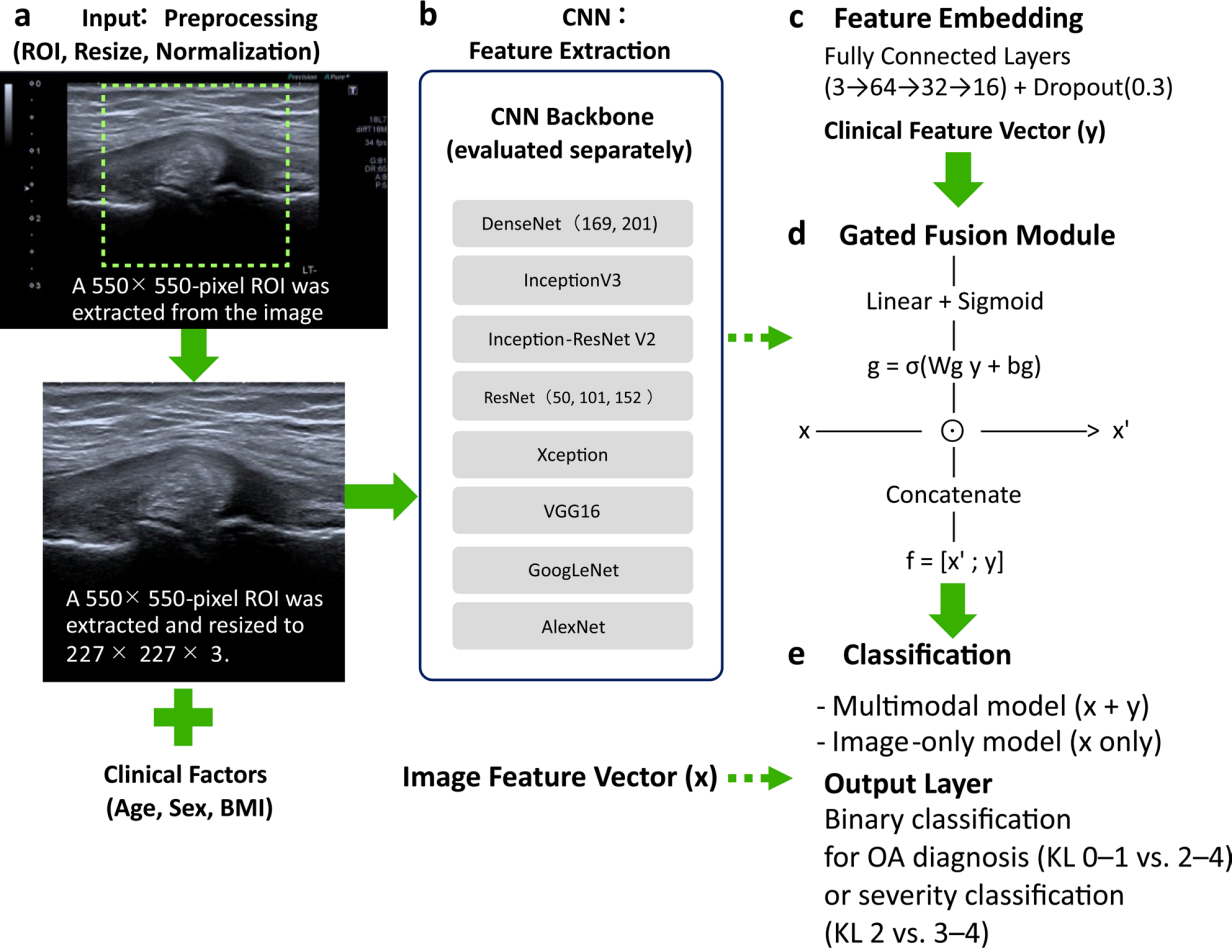
Multimodal CNN architecture for knee osteoarthritis diagnosis and severity assessment. This figure shows the overall architecture of the proposed multimodal CNN model. (**A**) Ultrasound images were preprocessed. (**B**) Image feature vectors (x) were extracted using multiple pretrained CNN backbones and evaluated independently. (**C**) Clinical features were embedded to generate a clinical feature vector (y). (**D**) A gated fusion module integrated image and clinical features. (**E**) The final classification was performed using either a multimodal or an image-only model to conduct binary classification for OA diagnosis (KL 0–1 vs. 2–4) or OA severity (KL 2 vs. 3–4)

The image-only model directly passed the feature vectors through the combined layers to the output. Age, sex, and BMI were input into the same fully connected layer and were standardized to ensure numerical consistency. Although sex is a binary variable, it was treated as a numerical feature rather than being one-hot encoded, in the same manner as the other continuous variables. The overall architecture of the proposed multimodal CNN model is illustrated in Fig. 1.

#### Model learning

To address class imbalance, focal loss (γ = 2.0, α = 0.7) was employed. The Adam optimizer was used with a learning rate of 1 × 10⁻⁴ to prevent excessive weight updates during fine-tuning of pretrained models. To mitigate overfitting, EarlyStopping (monitor = val_loss, patience = 10) and ReduceLROnPlateau (factor = 0.7, patience = 5, min_lr = 1 × 10⁻⁵) were applied.

Batch sizes of 2, 4, 8, and 16 were evaluated in preliminary experiments. Model performance was compared based on validation AUC and training time. Based on these results, and after confirming that performance was not strongly dependent on a specific batch size, a configuration achieving a favorable balance between performance and computational efficiency was selected for the final model.

Image data augmentation was applied to the training data as follows: random rotation ± 10°, translation up to ± 4 pixels each (approximately ± 1.8% of the image size), random zoom of 0.8–1.2 times, shear deformation within a maximum range of 20%, and left–right inversion with a probability of 50%. The blank areas outside the image were colored black (cval = 0). Model training was performed using five-fold cross-validation on the training/validation dataset. In each fold, the model was trained on the training subset and evaluated on the corresponding validation subset to monitor the learning process. The five models trained across the folds were retained, and the final performance evaluation was conducted on an independent test dataset that was not used during training. Ensemble inference was performed by averaging the predicted probabilities from the five models. The test dataset was not used for model training or model selection. All CNN architectures were trained under identical learning conditions, including the same optimizer, learning rate, loss function, batch size selection procedure, and training strategy, to ensure a fair comparison across models.

#### Performance evaluation and statistical analysis

The diagnostic performance of the models was evaluated using the AUC, sensitivity, specificity, PPV, NPV, F1 score, and diagnostic accuracy. Each index was calculated from the confusion matrix of the binary classification based on the cutoff value on the receiver operating characteristic (ROC) curve, which maximized the Youden Index (sensitivity + specificity – 1). The cutoff value was determined using the validation data from each fold of the cross-validation performed on the training dataset and was subsequently applied to the independent test dataset for performance evaluation. Statistical significance was set at *p* < 0.05. These analyses were performed using Python (TensorFlow, scikit-learn, and SciPy).

#### Statistical analysis of patient background

The background characteristics (age, sex, and BMI) of the participants in the OA and non-OA groups were compared. The Kolmogorov–Smirnov and F-tests were used to assess the normality and variance of each distribution. Student’s t-test was used to test for group differences if the distributions were normal and had equal variances. The chi-squared or Fisher’s exact test was used for categorical data analysis based on the expected frequency. Youden’s index was used to determine the cutoff values. Statistical analysis was performed using EZR (R version 2.4-0), and statistical significance was set at *p* < 0.05.

#### Assessing the importance of background factors using SHapley Additive exPlanations (SHAP)

SHAP was used to visualize the contributions of background characteristics to the model output [39]. Only the numerical input branches of the trained multimodal model were extracted and reconstructed using Keras. The data of 50 cases were selected from the independent test data for inclusion in the SHAP analysis. Those of 100 other cases were used to assess the distribution of the background data. The mean SHAP value for each factor was calculated from the values obtained using DeepExplainer to visualize feature importance.

#### Visualization of features using gradient-weighted Class Activation Mapping

Gradient-weighted Class Activation Mapping (Grad-CAM) was used to identify the visual regions used for the predictions of the image model. Grad-CAM is a method for visualizing important regions in an input image as a heatmap using the output of the final convolutional layer of a CNN and the gradient of the prediction score [40]. The outputs of the final convolutional layers of the CNN architectures and the gradients of the outputs were obtained for representative observations (including non-OA, OA, and severe OA) extracted from the test data using tf.GradientTape. The feature maps and gradients were globally averaged, and weighted synthesis was performed to create a Grad-CAM heat map. The generated Grad-CAM heat maps were overlaid on the original images to facilitate the visual evaluation of the correspondence of each model to the anatomical structures of interest (including joint crevices, bone surfaces, and synovium). Visual confirmation was performed by an orthopedic surgeon.

## Results

### Cases

**Fig. 2:**
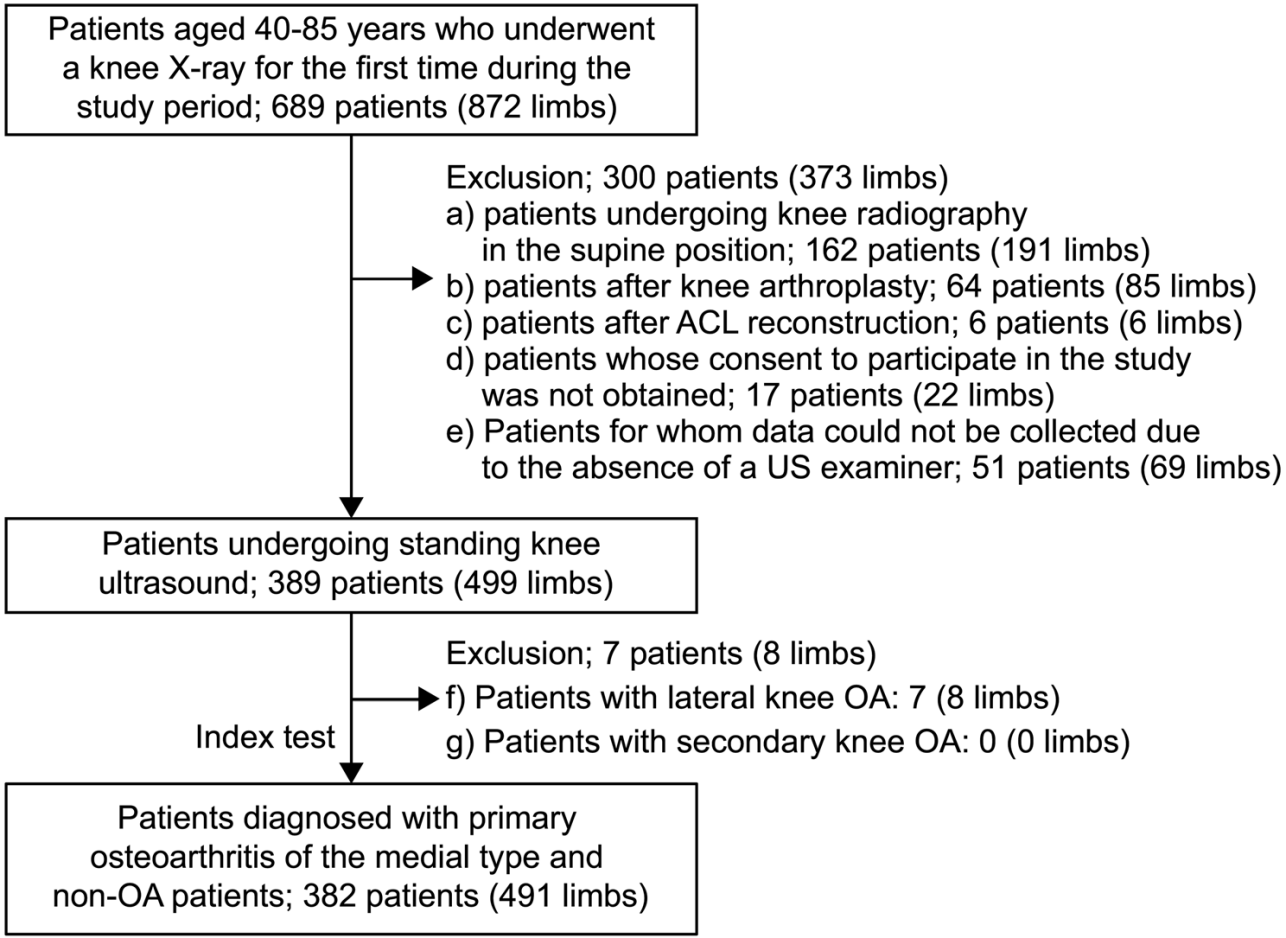
Flowchart of case selection. ACL, anterior cruciate ligament; OA, osteoarthritis; US, ultrasonography. Used with permission from Elsevier, from “Ultrasound-based radiomics and machine learning for enhanced diagnosis of knee osteoarthritis” [30], European Journal of Radiology Open, Vol. 14, 2025; permission conveyed through the Copyright Clearance Center, Inc

Figure 2 presents a flowchart of the study methodology. The study initially enrolled 872 limbs of the 689 cases. The data of the patients who underwent supine knee radiography (162 cases and 191 limbs), those who had knee replacement (64 cases and 85 limbs), those who had undergone reconstructive surgery (6 cases and 6 limbs), those who did not consent to participate (17 cases and 22 limbs), and those whose data could not be collected owing to the unavailability of an ultrasonographic examiner (51 cases and 69 limbs) were excluded before the ultrasonographic examination. The remaining 389 patients with 499 limbs underwent ultrasonographic examination. Seven patients (eight limbs) with lateral-type knee OA were excluded after ultrasonographic examination. Data for 491 limbs from 382 patients were included. The limbs of 318 and 173 participants were included in the clinical OA and non-OA groups, respectively. Statistical analysis confirmed the normal distribution and equal variance of age and BMI. Therefore, Student’s t-test was used to compare the groups. The chi-squared test was used to assess sex distribution because the expected frequency criteria were satisfied. Although cases with missing data were planned to be excluded, no missing values were observed in the cases ultimately included in the analysis.

### Patient background data stratified by the presence or absence of clinical OA

#### Age

The mean ages of patients in the clinical OA and non-OA groups were significantly different at 72.7 ± 8.7 and 62.6 ± 11.3 years, respectively (*p* < 0.001).

#### Sex distribution

The clinical OA group included 81 (25.5%) and 237 (74.5%) limbs, whereas the non-OA group included 73 (42.2%) and 100 (57.8%) limbs of male and female patients, respectively. This difference in sex distribution was significant (*p* < 0.001).

#### BMI

The mean BMI of patients in the clinical OA group (24.84 ± 3.89 kg/m²) was significantly higher than that of the non-OA group (23.47 ± 3.33 kg/m²) (*p* < 0.001). Table 2 compares the patient backgrounds.

**Table 2:** Comparison of the background data of patients in the clinical OA and non-OA groups

Variables	Clinical OA (*n* = 318)	Non-OA (*n* = 173)	*p*-value	All patients (*n* = 491)
Age	72.7 ± 8.7	62.6 ± 11.3	<0.001	69.1 ± 8.3
Sex			<0.001	
Male, n (%)	81 (25.5%)	73 (42.2%)		154 (31.4%)
Female, n (%)	237 (74.5%)	100 (57.8%)		337 (68.6%)
BMI	24.84 ± 3.89	23.47 ± 3.33	<0.001	24.36 ± 4.94

Data are expressed as mean ± standard deviation or value (%). BMI, body mass index; OA, osteoarthritis

Used with permission from Elsevier, from “Ultrasound-based radiomics and machine learning for enhanced diagnosis of knee osteoarthritis,” [30] European Journal of Radiology Open, Vol. 14, 2025; permission conveyed through the Copyright Clearance Center, Inc.

### Diagnostic performance of the CNN architectures used for the clinical OA diagnostic models (classification of OA presence/absence)

Comparison of image-only and multimodal models Table 3 shows the diagnostic performances of the image-only and multimodal models.

**Table 3:** Diagnostic performance of different CNN architectures for OA

Model	AUC	Diagnostic accuracy	Sensitivity	Specificity	PPV	NPV	F-value
**Estimated data (image-only)**							
DenseNet169	0.86	0.77	0.69	0.92	0.94	0.62	0.80
DenseNet201	0.86	0.77	0.71	0.89	0.92	0.62	0.80
InceptionV3	0.90	0.82	0.76	0.92	0.95	0.68	0.84
Inception-ResNet V2	0.84	0.76	0.72	0.85	0.90	0.62	0.80
ResNet50	0.82	0.80	0.80	0.79	0.88	0.68	0.84
ResNet101	0.87	0.78	0.69	0.94	0.96	0.62	0.80
ResNet152	0.90	0.82	0.74	0.96	0.97	0.67	0.84
Xception	0.87	0.79	0.79	0.79	0.87	0.67	0.83
VGG16	0.89	0.80	0.75	0.89	0.92	0.66	0.83
GoogLeNet	0.89	0.79	0.70	0.96	0.97	0.63	0.81
AlexNet	0.87	0.76	0.69	0.89	0.92	0.61	0.79
**Estimated data (multimodal)**							
DenseNet169	0.86	0.78	0.67	0.98	0.98	0.61	0.80
DenseNet201	0.88	0.80	0.77	0.85	0.90	0.67	0.83
InceptionV3	0.89	0.82	0.80	0.87	0.92	0.70	0.86
Inception-ResNet V2	0.84	0.81	0.76	0.90	0.94	0.67	0.84
ResNet50	0.85	0.72	0.59	0.96	0.97	0.56	0.74
ResNet101	0.89	0.84	0.83	0.85	0.91	0.73	0.87
ResNet152	0.88	0.83	0.77	0.94	0.96	0.69	0.86
Xception	0.87	0.77	0.67	0.96	0.97	0.61	0.79
VGG16	0.90	0.82	0.80	0.85	0.91	0.70	0.85
GoogLeNet	0.88	0.81	0.77	0.88	0.93	0.68	0.84
AlexNet	0.89	0.78	0.72	0.90	0.93	0.64	0.81

AUC, area under the curve; CNN, convolutional neural network; NPV, negative predictive value; OA, osteoarthritis; PPV, positive predictive value

Comparison among CNN architectures.

**Fig. 3:**
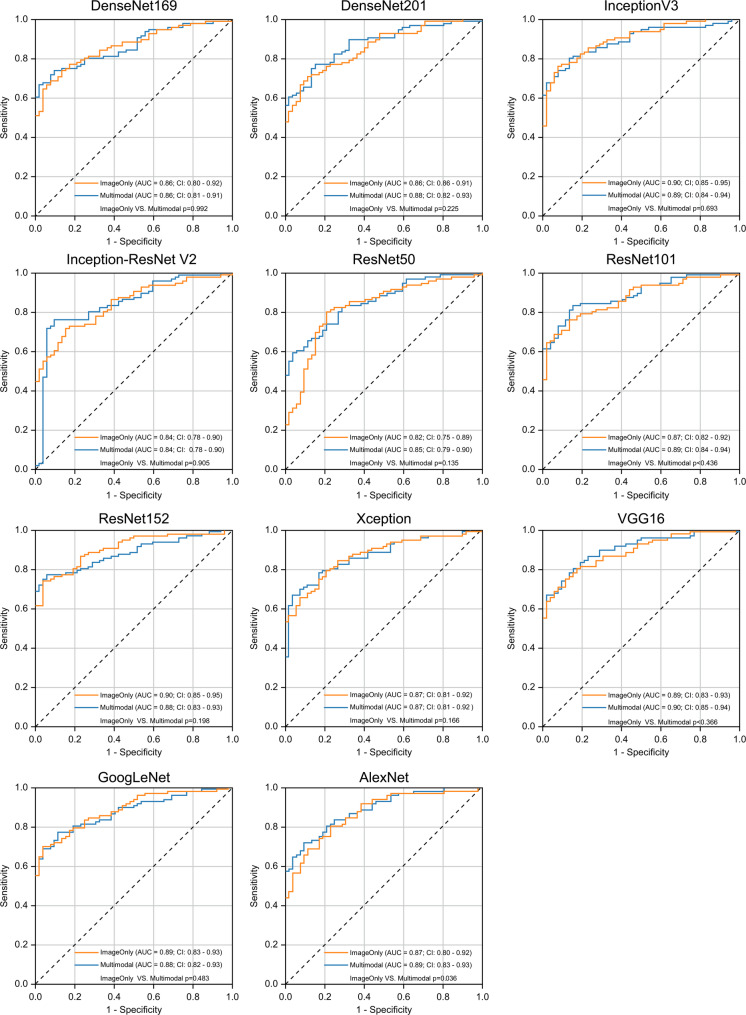
Comparison of the ROC curves for the different CNN architectures used for the OA diagnostic models. Orange line: ROC curve of the image-only model. Blue line: ROC curve of the multimodal model (background factors: age, sex, and BMI). AUC, area under the curve; BMI, body mass index; CI, confidence interval; CNN, convolutional neural network; ROC, receiver operating characteristic

Figure 3 presents ROC curves comparing the diagnostic performances of the image-only and multimodal models, as well as those of the different CNN architectures.

#### Metrics for the image-only model

The AUC ranged from 0.82 to 0.90 and was highest for InceptionV3 and ResNet152. Sensitivity ranged from 0.69 to 0.80 and was highest for ResNet50. Specificity ranged from 0.79 to 0.96 and was highest for ResNet152 and GoogLeNet. The PPV ranged from 0.87 to 0.97 and was highest for ResNet152 and GoogLeNet. The NPV ranged from 0.61 to 0.68 and was highest for InceptionV3 and ResNet50. The F1 score ranged from 0.79 to 0.84 and was highest for InceptionV3, ResNet50, and ResNet152.

#### Metrics for the multimodal model

The AUC ranged from 0.84 to 0.90 and was highest for VGG16. The sensitivity ranged from 0.59 to 0.83 and was highest for ResNet101. The specificity ranged from 0.85 to 0.98 and was highest for DenseNet169. The PPV ranged from 0.90 to 0.98 and was highest for DenseNet169. The NPV ranged from 0.56 to 0.73 and was highest for ResNet101. The F1 score ranged from 0.74 to 0.87 and was highest for ResNet101.

### Evaluation of background factor contribution using SHAP Analysis

**Fig. 4:**
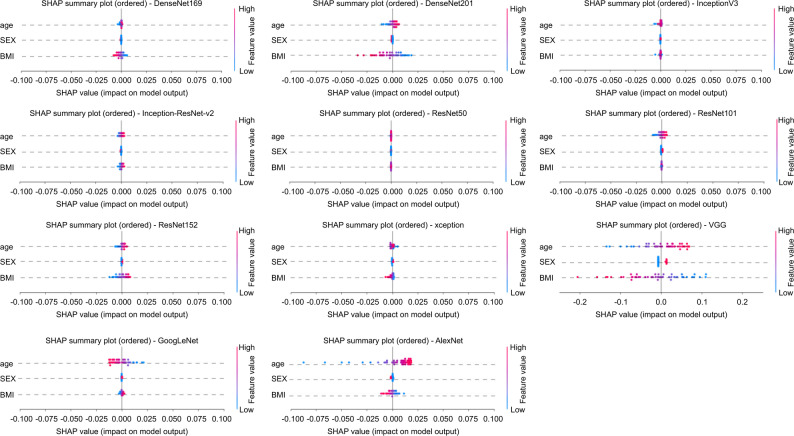
SHAP summary plot for each CNN architecture in the OA diagnostic model. The influence of age, sex, and BMI on the model output (SHAP values) is shown. This figure illustrates the contributions of age, BMI, and sex to the diagnosis of knee osteoarthritis. The horizontal axis represents the SHAP value, where positive values indicate contributions toward the OA class and negative values indicate contributions toward the non-OA class. The color represents the magnitude of each feature, with red indicating higher values and blue indicating lower values. Features are arranged in descending order based on their mean SHAP values. Across all CNN models, the mean SHAP values for age ranged from − 0.000889 to 0.005326, while those for sex ranged from − 0.000862 to 0.000048. The SHAP values for BMI ranged from − 0.01367 to 0.002366. SHAP, SHapley Additive exPlanations. CNN, convolutional neural network; ROC, receiver operating characteristic; OA, osteoarthritis

The SHAP summary plots of age, sex, and BMI for each CNN model are presented in Fig. 4. The horizontal axis represents the SHAP value, where positive values indicate contributions toward the OA class and negative values indicate contributions toward the non-OA class. The color represents the magnitude of each feature, with red indicating higher values and blue indicating lower values. Features are arranged in descending order based on their mean SHAP values. The mean SHAP values for age ranged from − 0.000889 to 0.005326, those for sex ranged from − 0.000862 to 0.000048, and those for BMI ranged from − 0.01367 to 0.002366. The mean SHAP values for age, sex, and BMI for each CNN model are listed in Additional file 3 Table S2.

### Comparison of patient backgrounds stratified by severity of clinical OA

Of the 318 patients with clinical OA, 117 and 201 had severe and mild limb deformities, respectively. Statistical analysis confirmed the normal distribution and homogeneity of variances of age and BMI. Therefore, Student’s t-test was used to compare the severe and mild deformity groups. The chi-squared test was used to compare the sex distributions because the frequencies met the criteria.

#### Age

The mean ages of patients in the severe and mild deformity groups were significantly different at 73.7 ± 8.5 and 72.1 ± 10.3 years, respectively. The age difference was not significant (*p* = 0.121).

#### Sex distribution

The severe deformity group included 29 (24.8%) and 88 (75.2%) limbs of male and female patients, respectively. The mild deformity group included 52 limbs (25.9%) of male patients and 149 limbs (74.1%) of female patients. The difference was not significant (*p* = 0.936).

#### BMI

The mean BMI of patients in the severe deformity group was 25.43 ± 4.09 kg/m², which was significantly higher than that of the mild deformity group (24.50 ± 3.74 kg/m²) (*p* = 0.039).

### Diagnostic performance of the CNN architectures used for the severe OA diagnostic models (severity classification)

Comparison of image-only and multimodal models.

Table 4 shows the diagnostic performance of the image-only and multimodal models.

**Table 4:** Diagnostic performance of CNN architectures for severity classification

Model	AUC	Diagnostic accuracy	Sensitivity	Specificity	PPV	NPV	F-value
**Estimated data (image-only)**							
DenseNet169	0.90	0.84	0.83	0.85	0.76	0.90	0.79
DenseNet201	0.91	0.86	0.89	0.85	0.78	0.93	0.83
InceptionV3	0.88	0.79	0.91	0.72	0.65	0.94	0.76
Inception-ResNet V2	0.84	0.76	0.86	0.71	0.63	0.90	0.72
ResNet50	0.87	0.77	0.89	0.89	0.79	0.87	0.78
ResNet101	0.73	0.65	0.86	0.52	0.51	0.86	0.64
ResNet152	0.80	0.73	0.86	0.66	0.59	0.89	0.70
Xception	0.89	0.81	0.89	0.77	0.69	0.92	0.78
VGG16	0.88	0.83	0.94	0.77	0.70	0.96	0.82
GoogLeNet	0.79	0.74	0.71	0.75	0.63	0.82	0.67
AlexNet	0.85	0.84	0.86	0.87	0.79	0.91	0.79
**Estimated data (multimodal)**							
DenseNet169	0.88	0.82	0.77	0.85	0.75	0.87	0.76
DenseNet201	0.88	0.84	0.80	0.87	0.78	0.88	0.79
InceptionV3	0.92	0.86	0.86	0.87	0.79	0.91	0.82
Inception-ResNet V2	0.90	0.82	0.89	0.79	0.72	0.92	0.78
ResNet50	0.90	0.81	0.91	0.75	0.71	1.00	0.83
ResNet101	0.79	0.73	0.71	0.74	0.61	0.82	0.66
ResNet152	0.83	0.79	0.71	0.84	0.71	0.84	0.71
Xception	0.91	0.84	0.91	0.80	0.73	0.94	0.81
VGG16	0.90	0.81	0.94	0.74	0.67	0.96	0.79
GoogLeNet	0.80	0.73	0.77	0.70	0.60	0.84	0.68
AlexNet	0.85	0.84	0.89	0.82	0.74	0.93	0.81

AUC, area under the curve; NPV, negative predictive value; PPV, positive predictive value

Comparison among CNN architectures.

**Fig. 5:**
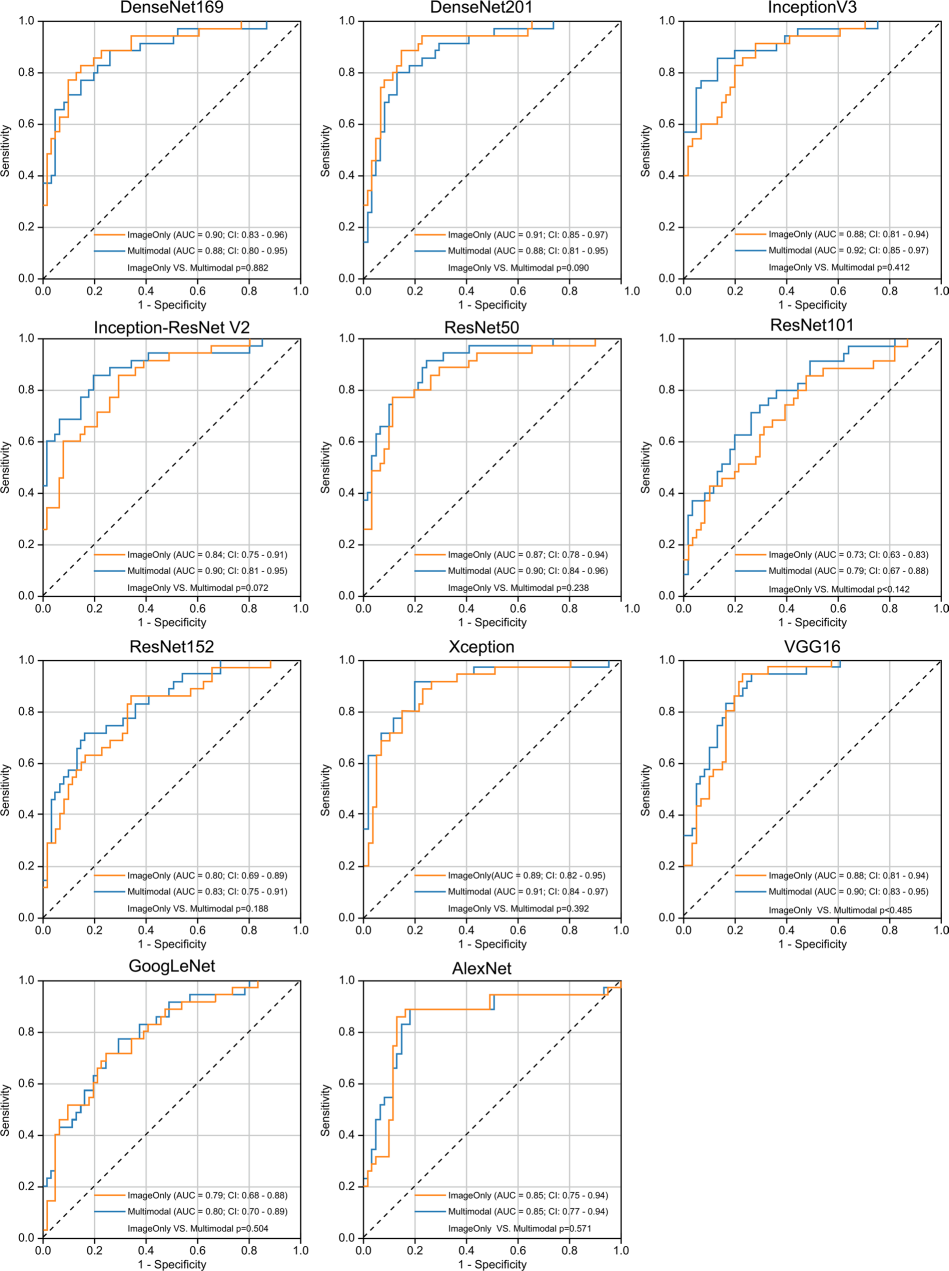
ROC curves for severe OA diagnosis models based on different CNN architectures. Orange line: ROC curve of the image-only model. Blue line: ROC curve of the multimodal model (background factor: BMI only). AUC, area under the curve; BMI, body mass index; CI, confidence interval; OA, osteoarthritis; ROC, receiver operating characteristic

Figure 5 presents ROC curves comparing the diagnostic performances of the image-only and multimodal models, as well as those of the different CNN architectures.

### Typical results for the image-only model

The AUC ranged from 0.73 to 0.91 and was highest for DenseNet201 and AlexNet. Sensitivity ranged from 0.71 to 0.94 and was highest for VGG16. The specificity ranged from 0.52 to 0.89 and was highest for ResNet50. The PPV ranged from 0.51 to 0.79 and was highest for ResNet50. The NPV ranged from 0.82 to 0.96 and was highest for VGG16. The F-score ranged from 0.64 to 0.83 and was highest for DenseNet201.

### Representative results of the multimodal model

The AUC ranged from 0.79 to 0.92 and was highest for InceptionV3. Sensitivity ranged from 0.71 to 0.94 and was highest for VGG16. Specificity ranged from 0.70 to 0.87 and was highest for DenseNet201 and InceptionV3. The PPV ranged from 0.60 to 0.79 and was highest for InceptionV3. The NPV ranged from 0.82 to 1.00 and was highest for ResNet50. The F-value ranged from 0.66 to 0.83 and was highest for ResNet50.

### Evaluation of background factor contribution using SHAP analysis

**Fig. 6:**
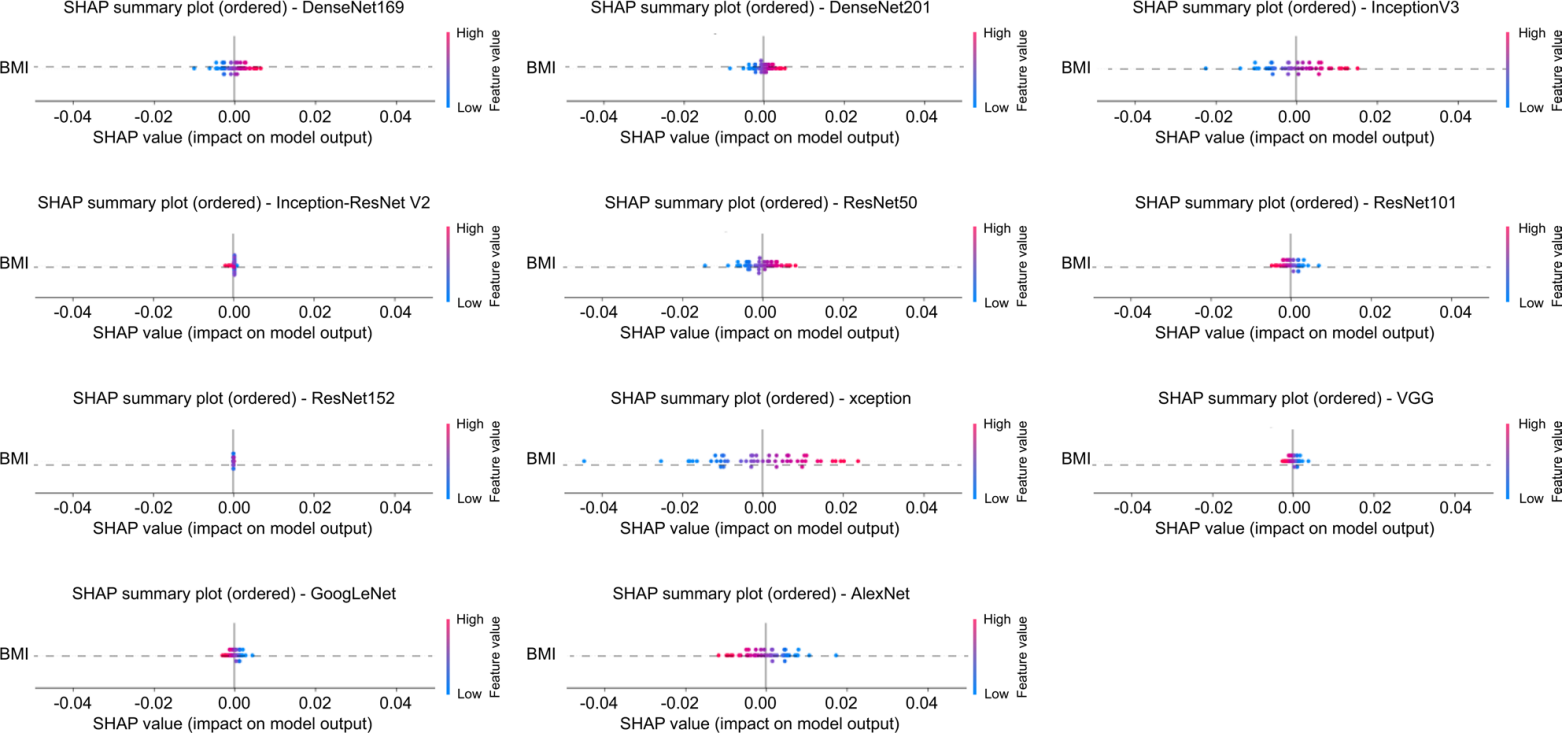
SHAP summary plot for each CNN architecture used for the severe OA diagnostic model. The influence of BMI on the model output (SHAP values) is shown. This figure illustrates the contribution of BMI to the diagnosis of knee osteoarthritis. The horizontal axis represents the SHAP value, where positive values indicate contributions toward the OA class and negative values indicate contributions toward the non-OA class. The color represents the magnitude of the feature, with red indicating higher values and blue indicating lower values. Features are arranged in descending order based on their mean SHAP values. Across all CNN models, the SHAP values for BMI ranged from − 0.00075 to 0.000341. SHAP, SHapley Additive exPlanations

The SHAP summary plots for BMI for each CNN model are shown in Fig. 6. The horizontal axis represents the SHAP value, where positive values indicate contributions toward the OA class and negative values indicate contributions toward the non-OA class. The color represents the magnitude of each feature, with red indicating higher values and blue indicating lower values. Features are arranged in descending order based on their mean SHAP values.

The SHAP values for BMI in the severity classification models ranged from − 0.00075 to 0.000341. The mean SHAP values for BMI for each CNN model are shown in Additional file 4 Table S3.

### Grad-CAM visualization of regions of interest by KL grade

**Fig. 7:**
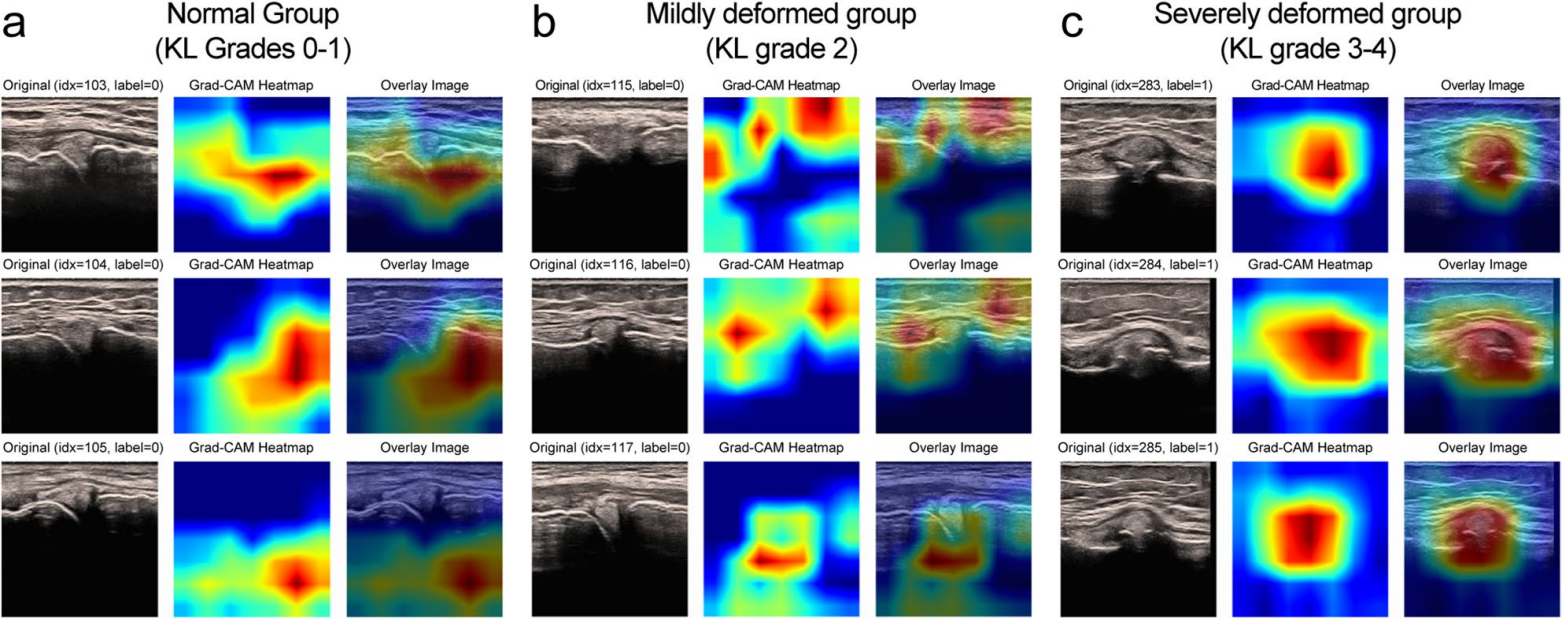
Representative Grad-CAM activation regions by KL grade. (**A**) Representative Grad-CAM heat maps for OA diagnosis in the non-OA group (KL grades 0–1). (**B**) Representative Grad-CAM heat maps for OA diagnosis in the mild OA group (KL grade 2). (**C**) Representative Grad-CAM heat maps for OA severity classification in the severe OA group (KL grades 3–4). From left to right, the panels show the original ultrasound image, the Grad-CAM heat map, and the heat map overlaid on the original image

The characteristics of the Grad-CAM regions of interest for each KL grade are shown in Fig. 7.

#### Normal Group (KL grades 0–1)

The regions of interest highlighted by Grad-CAM generally had weak activation and mainly colored blue and green. They were also low attention areas; attention was focused near the subcutaneous tissue and fascia in the superficial layer but not on the joint crevices or bony surfaces.

#### Mildly deformed group (KL grade 2)

Areas of increased uptake were observed in the joint crevice and around the tibial articular surface. However, the distribution and intensity of uptake varied among the cases.

#### Severely deformed group (KL grades 3–4)

The area of interest was clear. Red and yellow areas of high interest were concentrated in the regions of joint crevice narrowing, osteophyte formation, and synovial thickening.

## Discussion

Early diagnosis of knee OA has important implications and provides an opportunity to intervene before structural damage progresses. A recent review of trends in the development of disease-modifying OA agents (DMOADs) reported irreversible damage to joint structures in advanced OA (KL grades 3 and 4). This limits the effectiveness of DMOADs. In contrast, the joint structure is partially preserved in early OA (KL grades 1 and 2), and structural improvement is possible with treatment. The effectiveness of DMOADs may be maximized for these [41]. Eleven DMOAD candidates have been evaluated in Phase II or III clinical trials, and six of them have demonstrated significant structural or symptomatic improvement. None of these candidates have been approved by the US Food and Drug Administration; however, clinical evidence for their use as therapeutic interventions is accumulating, and the development of DMOADs is entering a new phase. Therefore, the establishment of early diagnostic techniques for OA will play a pivotal role in future preventive interventions and the implementation of disease-modifying therapies. In patients with KL grade 3 or higher knee OA, the effectiveness of conservative treatment is often limited, and surgical intervention is commonly considered at this stage. Previous studies on indications for total knee arthroplasty have demonstrated that, in addition to severe pain and functional impairment, radiographically advanced structural changes constitute an important determinant in clinical decision-making, with disease severity corresponding to KL grade 3 or higher being one of the criteria for considering surgical treatment [42]. Furthermore, patients with KL grade 3 knee OA have been reported to achieve functional improvements after total knee arthroplasty comparable to those observed in patients with KL grade 4 disease, suggesting that KL grade 3 represents a clinically meaningful threshold at which surgical treatment may be considered [43]. Taken together, these findings indicate that distinguishing between KL grade 2 and KL grades 3–4 is clinically important for determining treatment strategies.

In this study, we developed CNN models using US images and a multimodal CNN model that integrates age, sex, and BMI to improve the diagnostic accuracy of knee OA. We compared the performance of these models with those of conventional visual evaluation methods, radiomics and machine learning models, and CNN models using X-rays and MRI. In the diagnosis of OA, only the multimodal model based on AlexNet (0.89; 95% CI: 0.83–0.93) showed a significantly higher AUC than the image-only model (0.87; 95% CI: 0.80–0.92) (*p* = 0.036). However, the differences in AUC (0.02), diagnostic accuracy (+ 0.02), sensitivity (+ 0.03), and F-score (+ 0.02) were minimal and of limited clinical significance. SHAP analysis showed that the mean SHAP values for age, sex, and BMI were all close to zero, indicating that the influence of these background factors on the model output was limited and that the model made predictions primarily based on image-derived information rather than background factors.

In the severity classification, BMI was a significant background factor and was incorporated into the multimodal CNN; however, no significant differences in AUC were observed among the architectures. SHAP analysis also showed that the mean SHAP values for BMI were all close to zero, suggesting that the model made predictions primarily based on image-derived information rather than background factors.

Several possible reasons exist for this. First, OA is a multifactorial disease that cannot be adequately explained by clinical factors alone, and the clinical factors added in this study (age, sex, and BMI) alone may not have been sufficient to improve diagnostic accuracy. Although the clinical factors (age, sex, and BMI) integrated in this study are strongly associated with the risk of developing knee OA, they are not indicators that directly explain the progression of KL classification [34, 44]. Previous studies have reported that psychological [45] factors, sarcopenia [46], and lifestyle and nutritional factors [47, 48] also contribute to OA symptoms and progression, suggesting that clinical factors alone may not sufficiently capture the complete picture.

Second, data factors (sample size and distribution) are considered. Because this study incorporates a single-institution and regional population dataset, the variation in age and BMI is relatively small. Consequently, limited non-image differences were available from which the model could learn, and background factors may have contributed less to the classification performance. Furthermore, when the information content of background factors is limited (e.g., on the scale of a few hundred examples), the “relative weight” of the non-image data is smaller than the rich feature set that the CNN extracts from the images, and the possibility that the data may be buried in image-derived signals, even after integration cannot be ruled out [44].

Third, technical factors (i.e., model design) may have influenced the results. The non-image data used for integration in this study was limited to only three variables, and the gated fusion mechanism, which assumes multidimensional data, may not have functioned adequately. Moreover, low-dimensional clinical data are easily buried in high-dimensional image features [49, 50], and the advantage of fusion in this study—“the effect of integrating multidimensional information to enhance patterns”—was not fully demonstrated.

In this study, we extended the analysis beyond a simple comparison of performance metrics and examined how the structural characteristics of each CNN model contribute to diagnostic performance. For OA screening (KL 0–1 vs. 2–4), high sensitivity and high NPV are particularly important. In this context, ResNet50 and Xception demonstrated high sensitivity (80% and 79%, respectively), indicating strong detection capability. These models benefit from residual connections and efficient feature propagation, enabling stable learning of deep representations and facilitating the detection of early OA-related changes.

In contrast, during the diagnostic confirmation stage, high specificity and PPV are required to minimize false-positive results. In our study, ResNet152 and GoogLeNet achieved high specificity (96–97%), indicating high reliability for positive diagnosis. In particular, GoogLeNet employs an Inception architecture that integrates convolutional filters with different receptive fields in parallel, allowing both local and global joint structures to be captured, which is advantageous for definitive diagnosis.

Furthermore, in the classification of severe OA (KL 3–4), VGG16 and InceptionV3 demonstrated high sensitivity and NPV, indicating their usefulness in minimizing missed severe cases. VGG16 provides stable hierarchical feature representations that effectively capture progressive morphological changes, whereas InceptionV3 enables multi-scale feature extraction suitable for evaluating extensive structural alterations associated with advanced disease.

**Fig. 8:**
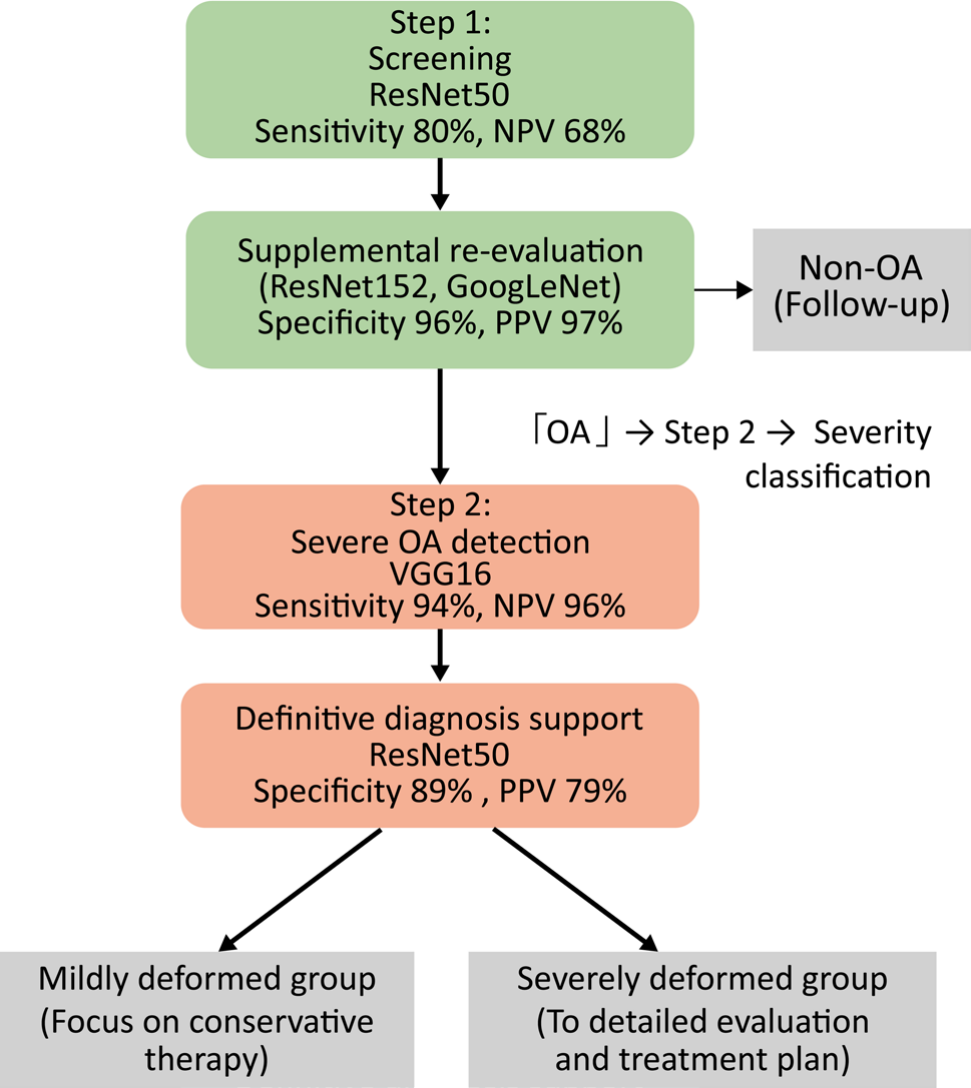
Flowchart for the diagnosis and severity classification of knee OA. OA, osteoarthritis

Based on these findings, we propose a two-step diagnostic workflow for knee OA (Fig. 8). In Step 1, a high-sensitivity model (ResNet50) is used to minimize missed OA cases, followed by supplementary re-evaluation using ResNet152 and GoogLeNet to reduce false-positive results. In Step 2, cases identified as OA undergo severity assessment, where VGG16 is applied to sensitively detect severe OA (KL 3–4). Additionally, ResNet50 supports diagnostic confirmation, thereby achieving a balance between diagnostic accuracy and clinical reliability.

Overall, our findings suggest an association between CNN architectural characteristics and diagnostic performance. These observations are consistent with previous reports describing residual learning in ResNet [51], hierarchical feature representations in VGG networks [52], and multi-scale feature extraction in Inception-based architectures [53]. However, this study does not establish a direct causal relationship between architectural design and performance, and the observed differences may also be influenced by dataset characteristics and optimization processes.

In this study, we aimed to apply the model to clinical diagnostic support and, therefore, focused on classification performance directly relevant to clinical decision-making. Accordingly, we adopted a binary classification scheme defining KL 0–1 as non-OA and KL 2–4 as OA. This criterion, based on the presence or absence of radiological changes, is expected to improve classification accuracy. The diagnostic accuracy for multiclass classification (five classes for KL grades 0–4) was generally low for all studies reviewed. Mohammed et al. [23] reported a diagnostic accuracy of 69.0% for the ResNet-101 model. Pi et al. [24] reported an accuracy of 76.9% for a CNN ensemble. Kim et al. [26] reported an accuracy of 51.9% for an SE-ResNet model based only on images and 61.6% when images and background factors were used for prediction. Tiulpin and Saarakkala [27] reported an accuracy of 66.7% for a ResNet ensemble. Antony et al. [54] reported an accuracy of 63.4% for a lightweight CNN, and Guida et al. [28] reported an accuracy of 54.0% for the 3D ResNet. Low sensitivity, especially for mild OA (KL grade 1/2), has also been reported. Tiulpin et al. [55] reported a sensitivity of 11.0% for KL grade 1, whereas Antony et al. [54] reported 15.0% and Guida et al. [28] reported 7.5%. Mohammed et al. [23] reported 48.2% and 59.7% for KL grades 1 and 2, respectively. This indicates that the risk of missing early OA is high. The comprehensive review by Piccolo et al. [56] on knee OA highlighted that AI models with AUCs and diagnostic accuracies of approximately 70% are in the exploratory research phase and must be improved for clinical application. They reported that these models are useful as support tools for diagnosing OA, which requires early interventions. Mohammed et al. [23] reported accuracies for the five- and three-class classification models. The three-class classification included KL grades 2, 3, and 4, denoting mild, moderate, and severe OA, respectively. The sensitivities for the three were 92.8%, 81.2%, and 88.2%, respectively. However, both KL grades 3 and 4 indicate advanced OA, and their treatment strategies are not significantly different. Therefore, a two-class setting that integrates them is more clinically practical. Mohammed et al. [23] reported diagnostic accuracies of 69.0% and 87.5% for the 5- and 2-class models, respectively. Guida et al. [28] also reported improvement from 54.0% to 83.1%, indicating a higher diagnostic accuracy for binary classification. The diagnostic performances of multiclass classification models reported in previous studies are summarized in Additional file 5 Table S4. The diagnostic performance and clinical usefulness of the binary classification model were also evaluated and compared with those reported in previous studies (Additional file 6 Table S5).

Yanagisawa et al. [17] reported a high diagnostic accuracy of conventional US with sensitivity and specificity of 91% and 96%, respectively. However, it is susceptible to alignment effects associated with the progression of knee OA, and reproducibility remains an issue. The TOH-DBB index reported by Kiso et al. [18] is stable and alignment independent, and its sensitivity and specificity are 80.0% and 76.0%, respectively. However, this method is limited to shallow structures. Radiomics-based nomogram models using radiological features and machine learning have demonstrated diagnostic performance for knee OA, with a reported sensitivity of 81.0% and specificity of 80% [20]. However, because these approaches rely on predefined handcrafted features as inputs, the feature space is inherently constrained, and the analysis is limited to this predefined domain. In addition, the requirement for manual ROI delineation introduces potential limitations in terms of efficiency and reproducibility.

In contrast, the CNN model employed in the present study automatically learns feature representations from entire images through end-to-end training. By not relying on predefined feature engineering, the model can learn optimal representations directly from the data, enabling it to capture high-dimensional and nonlinear information that may not be adequately represented by conventional radiomics approaches. The present study, therefore, proposes a fundamentally different analytical framework from traditional feature-based methods and demonstrates the potential utility of an integrated deep learning-based diagnostic model.

Mohammed et al. [23] and Tiulpin et al. [55] reported a high diagnostic accuracy of X-ray-based CNNs; however, they are limited by radiation exposure. Conversely, MRI-based CNNs have high diagnostic accuracies [28]. Yeoh et al. [57] reported good results for them, but they are expensive and not portable. They also make it difficult to capture joint changes that become apparent during loading because they use non-loaded imaging. US can be used to complementary tool to X-ray and MRI to address their disadvantages because it is free of radiation exposure and allows real-time and weight-bearing evaluation.

In this study, Grad-CAM was adopted to visualize the decision-making process of the CNN. Grad-CAM has been widely used in medical image analysis and offers a favorable balance between interpretability and reproducibility, facilitating comparison with previous studies. Recently, transformer-based models such as Vision Transformer and high-resolution visualization techniques have been proposed; however, these approaches often require large-scale datasets and have been reported to be prone to overfitting when applied to relatively small datasets [58, 59]. The primary objective of this study was not to achieve precise pixel-level localization, but rather to capture the overall anatomical regions contributing to diagnostic decision-making. Therefore, from the perspectives of computational efficiency and generalizability, Grad-CAM was considered an appropriate choice for this study. Nevertheless, HiResCAM has the potential to provide more fine-grained spatial information and represents a promising approach for future investigations.

For severe OA, attention was focused on the narrowing of the joint cleft and osteophyte formation. This suggests that model judgments based on structural changes can be visualized. Moreover, the regions of interest for the normal to mild disease group were distributed throughout the image rather than being concentrated in characteristic abnormal areas, such as joint fissures and osteophytes. This pattern reflected the inability of a model to classify cases with few structural abnormalities. The term “unclear diagnostic basis” is used when the diagnosis is not clear from the images. CAM can also help identify cases with unreliable diagnoses by visually indicating uncertainty and encouraging physicians to reconfirm or reconcile diagnoses with other diagnostic information.

In the present study, multimodal models integrating age, sex, and BMI did not consistently improve performance across all CNN architectures, and the performance gain was limited except for the AlexNet model. This finding indicates that multimodal integration does not necessarily lead to improved diagnostic performance in all cases, representing an important insight.

One possible explanation is that recent deep learning models possess a high capacity for feature representation and are able to implicitly capture clinical characteristics, such as age and body composition, from imaging data alone. Previous studies have demonstrated that high-performing CNNs can infer non-imaging attributes from visual patterns [60, 61], which may reduce the incremental benefit of explicitly incorporating background variables. Consequently, the addition of such variables may yield only limited performance gains in models with strong representational capacity.

Furthermore, this finding is consistent with that of previous X-ray- and MRI-based studies reporting that CNN models trained using imaging data alone can achieve high diagnostic performance, while the addition of background factors such as age, sex, and body mass index results in limited or inconsistent performance improvements [25, 26]. Taken together, these reports suggest that CNNs with high representational capacity may implicitly encode clinically relevant characteristics within imaging features themselves, potentially explaining the limited effect of multimodal integration observed in the present study.

In contrast, the observation that multimodal integration improved performance in AlexNet, which has a relatively limited representational capacity, suggests that the contribution of background variables may depend on the expressive power of the model. This finding implies that the effectiveness of multimodal learning is influenced by model architecture and capacity.

Taken together, our results indicate that multimodal integration does not universally guarantee performance improvement and that its effectiveness varies depending on model characteristics and task complexity. These findings highlight the importance of carefully considering the compatibility between model architecture and input information when designing diagnostic models for knee OA.

This study has some limitations. First, it was a single-center study; therefore, diversity in background characteristics such as age and body composition may not have been sufficiently represented. To evaluate the generalizability of the proposed model, future validation using multicenter datasets that include diverse patient populations is required. Multicenter collaborative studies may allow the model to learn from a broader spectrum of cases, thereby potentially improving its robustness and clinical applicability. Second, only age, sex, and BMI were used as background variables in this study, as these represent basic information that is routinely and easily obtainable in clinical practice. Therefore, the present study does not aim to comprehensively evaluate the overall usefulness of structured clinical data, but rather to assess the impact of integrating a limited set of clinical variables on diagnostic performance. In the future, incorporating multidimensional data such as pain scores, gait function, and activity limitations may enable the development of diagnostic support models that more accurately reflect real-world clinical conditions. Third, in the present study, the left and right knees of the same patient were treated as independent samples, and data splitting was performed at the knee level. Previous studies have reported that although the presence of OA in one knee constitutes a risk factor for OA development in the contralateral knee, disease progression does not necessarily occur simultaneously or to the same extent in both knees, with differences observed in the timing of onset and disease severity between sides [62]. In addition, ultrasonography-based studies of knee OA commonly assess disease severity by treating each knee as an independent evaluation unit, adopting study designs in which the left and right knees are analyzed as separate cases [15]. Furthermore, recent image analysis and deep learning studies have also adopted a knee-level analytical approach, in which each knee is treated as a single sample and bilateral knees from the same subject are independently used for model training and evaluation [55]. Based on these findings, we treated the left and right knees as independent analytical units in the present study. However, particularly in multimodal models integrating background factors, information shared at the subject level may be included across data splits, potentially leading to information leakage. In contrast, in models based solely on imaging data, this effect is considered to be relatively limited because anatomical and pathological differences exist between the left and right knees. This issue was explicitly recognized as a limitation of the present study, and the results were interpreted with caution. Future studies should further evaluate the generalizability of the proposed models by performing subject-level data splitting and/or incorporating external validation datasets. Fourth, there is a limitation regarding the acquisition of ultrasound images: US is inherently highly operator-dependent, and as there are not enough internationally unified guidelines for knee OA diagnosis, the accuracy of image quality may depend on the examiner’s experience and skill. In particular, the absence of standardized guidelines may lead to variations in probe positioning, angle, and pressure among examiners, which could affect image consistency and diagnostic accuracy. In this study, the existing standardization method for image acquisition was conducted according to a predefined institutional protocol, and Inter-examiner error was evaluated in advance to confirm high reproducibility, as well as to verify the examiner’s diagnostic ability. Although these measures are believed to have minimized the impact of examiner dependence on image quality, it is difficult to eliminate it completely [18]. Finally, this study mainly focused on medial-type OA, and we were unable to examine differences in diagnostic accuracy and characteristics of each joint site, such as the medial, lateral, and patellofemoral joints. This represents another limitation of this study, and future evaluation of model performance should consider the characteristics of each site.

Despite these limitations, this study has several notable strengths. First, the use of a standardized US acquisition protocol ensured consistent image quality and minimized inter-operator variability, thereby enhancing the reliability of deep learning analysis. Second, this study demonstrated the diagnostic utility of CNN models trained on US images and clarified how the integration of background factors affects diagnostic performance, representing a meaningful contribution to musculoskeletal imaging research. Third, the application of explainable AI techniques, such as Grad-CAM and SHAP, improved the interpretability and transparency of AI-assisted diagnosis and provided educational value for clinicians and researchers. In addition, this study holds practical significance: US-based AI models may serve as cost-effective and accessible diagnostic tools for knee OA, particularly in healthcare settings where advanced imaging modalities are not readily available. Overall, the study findings highlight the potential applicability and clinical relevance of AI-assisted ultrasonography in musculoskeletal diagnoses.

## Conclusions

In this study, we developed and evaluated two types of CNN-based models for the diagnosis and severity classification of knee OA: an image-only model using US images and a multimodal model that integrated background factors such as age, sex, and BMI. No substantial differences in performance were observed between these models, suggesting that US imaging alone provides sufficient information to support diagnostic assistance for knee OA. In particular, this approach may be beneficial for primary care physicians by enabling early identification of OA without reliance on radiographs. As a clinical screening and diagnostic support tool, it may contribute to clinical decision-making, improve workflow efficiency, and facilitate earlier patient management, especially in settings with limited access to advanced imaging modalities.

## Supplementary Information

Below is the link to the electronic supplementary material.

Supplementary Material 1: File name: Additional file 1 Figure S1. File format: .docx. Title of data: Probe positioning and corresponding ultrasound image of the medial knee joint. Description of data: This figure shows probe positioning relative to the medial femoral epicondyle and corresponding long-axis ultrasound image of the medial knee joint, including identification of key anatomical landmarks

Supplementary Material 2: File name: Additional file 2 Table S1. File format: .docx. Title of data: Diagnostic accuracy of CNN models in prior studies. Description of data: This table summarizes the diagnostic performance metrics of CNN models reported in previous studies, allowing comparison with those of the present study

Supplementary Material 3: File name: Additional file 3 Table S2. File format: .docx. Title of data: SHAP values for age, sex, and BMI for each CNN model. Description of data: This table lists the SHAP values for age, sex, and BMI across all CNN models, illustrating the contribution of demographic variables to the prediction output

Supplementary Material 4: File name: Additional file 4 Table S3. File format: .docx. Title of data: SHAP values for BMI for each CNN model. Description of data: This table summarizes the SHAP values representing the contribution of BMI to the prediction output for each CNN model

Supplementary Material 5: File name: Additional file 5 Table S4. File format: .docx. Title of data: Results of previous studies on multiclass classification. Description of data: This table provides a summary of previous studies presenting multiclass classification for knee osteoarthritis, including target classes, methods, and performance metrics

Supplementary Material 6: File name: Additional file 6 Table S5. File format: .docx. Title of data: Comparison of the diagnostic accuracies of the two classifications. Description of data: This table compares diagnostic accuracy of two classification strategies (KL 0–1 vs. 2–4 and KL 0 vs. 1–4), highlighting differences in sensitivity, specificity, and overall performance

## Data Availability

The datasets used and/or analyzed during the current study are available from the corresponding author on reasonable request.

## Permission to reproduce material from other sources

Permission already obtained.

In accordance with Elsevier’s policy, permission to reuse the following table and figure has been obtained via the RightsLink system.

Reference:

Kiso T, Okada Y, Kawata S, et al. [20].

Materials for which permission has been obtained: Table 1: Comparison of the background of patients in the clinical OA and non-OA groups. Figure 3: Flowchart of case selection.

## Abbreviations

AUC: Area under the curve
BMI: Body mass index
CNN: Convolutional neural network
KL: Kellgren–Lawrence
MRI: Magnetic resonance imaging
NPV: Negative predictive value
OA: Osteoarthritis
PPV: Positive predictive value
ROC: Receiver operating characteristic
ROI: Region of interest
US: Ultrasonography
