# Supplementary material for: Multimodal model integrating ultrasound and demographic data for the diagnosis of knee osteoarthritis
Source: BMC Med Imaging. 2026 Apr 2;26:248. doi: 10.1186/s12880-026-02249-8 (PMC13169557; doi:10.1186/s12880-026-02249-8)
Supplement: Supplementary file 1 — Supplementary Material 1: File name: Additional file 1 Figure S1. File format: .docx. Title of data: Probe positioning and corresponding ultrasound image of the medial knee joint. Description of data: This figure shows probe positioning relative to the medial femoral epicondyle and corresponding long-axis ultrasound image of the medial knee joint, including identification of key anatomical landmarks [file 12880_2026_2249_MOESM1_ESM.docx]

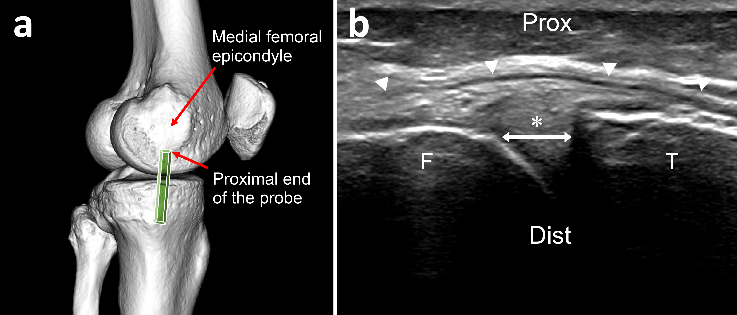


**Additional file Figure S1. Probe positioning and corresponding ultrasound image of the medial knee joint.**

(a) Probe positioning with reference to the medial femoral epicondyle.

(b) Long-axis ultrasound image of the medial joint cleft.

F: femur; T: tibia; Prox: proximal; Dist: distal; *: medial meniscus; arrowheads: medial collateral ligament; white double arrow: joint cleft
