# Supplementary material for: Multimodal model integrating ultrasound and demographic data for the diagnosis of knee osteoarthritis
Source: BMC Med Imaging. 2026 Apr 2;26:248. doi: 10.1186/s12880-026-02249-8 (PMC13169557; doi:10.1186/s12880-026-02249-8)
Supplement: Supplementary file 2 — Supplementary Material 2: File name: Additional file 2 Table S1. File format: .docx. Title of data: Diagnostic accuracy of CNN models in prior studies. Description of data: This table summarizes the diagnostic performance metrics of CNN models reported in previous studies, allowing comparison with those of the present study [file 12880_2026_2249_MOESM2_ESM.docx]

**Additional file Table S1**. Diagnostic accuracy of CNN models in prior studies

| **Classification** | **Author** | **Target site/disease** | **Number of cases (n)** | **Model used** | **Representative model and results** |
| --- | --- | --- | --- | --- | --- |
| **US x orthopedic region** | Burlina et al. (2017) [1] | Diagnosis of myositis | 80 cases (33 in the non-myositis group + 47 in the myositis group) | **AlexNet** | Accuracy, 76.2%; Sensitivity, 81.6%; Specificity, 68.6% |
|  | Sasaki et al. (2024) [2] | Diagnosis of osteochondritis dissecans of the elbow | 158 cases (group without OCD: 91 cases + group with OCD: 67 cases) | **VGG16** | AUC, 0.95; Accuracy, 89.2%; Sensitivity, 94.0%; F1 score, 88.5% |
| **US x Non-orthopedic area** | Koh et al. (2020) [3] | Classification of thyroid nodules as benign or malignant | 13,560 cases (6,400 in the benign group + 7,160 in the malignant group) | **ResNet50** | AUC, 0.91; Accuracy, 83.4%; Sensitivity, 83.3%; Specificity, 83.5% |
|  |  |  |  | **InceptionResNetV2** | AUC, 0.92; Accuracy, 84.5%; Sensitivity, 84.0%; Specificity, 84.7% |
|  | Wu et al. (2022) [4] | Prediction of recurrence of hepatocellular carcinoma | 513 cases (220 in the non-recurrence group + 293 in the recurrence/death group) | **ResNet-18** | Prediction of early recurrence: AUC, 0.69  Prediction of late recurrence: AUC, 0.65 Recurrence-free survival: AUC, 0.61 |
|  | Zhou et al. (2020) [5] | Prediction of lymph node metastasis in breast cancer | 834 cases (414 in the group without lymph node metastasis + 420 in the group with lymph node metastasis) | **Inception-V3** | AUC, 0.89; Sensitivity, 85.0%; Specificity, 73.0%; F1 score, 81.0% |
|  |  |  |  | **ResNet-101** | AUC, 0.86; Sensitivity, 73.0%; Specificity, 73.0%; F1 score, 73.0% |
|  | Hu et al. (2023) [6] | Differentiation of ovarian disease | 202 cases (120 in the endometriotic cyst group + 82 in the ovarian abscess group) | **ResNet-152** | AUC, 0.99; Accuracy, 96.8%; Sensitivity, 90.0%; Specificity, 100.0%; F1 score, 94.7% |
|  |  |  |  | **DenseNet-161** | AUC, 0.92; Accuracy, 90.3%; Sensitivity, 90.0%; Specificity, 90.5%; F1 score, 85.7% |

AUC, area under the curve; CNN, convolutional neural network

**Additional References**

1. Burlina P, Billings S, Joshi N, Albayda J. Automated diagnosis of myositis from muscle ultrasound: exploring the use of machine learning and deep learning methods. PLOS One. 2017;12:e0184059. doi: [10.1371/journal.pone.0184059](https://doi.org/10.1371/journal.pone.0184059).

2. Sasaki K, Fujita D, Takatsuji K, Kotoura Y, Minami M, Kobayashi Y, et al. Deep learning-based osteochondritis dissecans detection in ultrasound images with humeral capitellum localization. Int J Comput Assist Radiol Surg. 2024;19:2143-52. doi: [10.1007/s11548-023-03040-8](https://doi.org/10.1007/s11548-023-03040-8).

3. Koh J, Lee E, Han K, Kim E-K, Son EJ, Sohn Y-M, et al. Diagnosis of thyroid nodules on ultrasonography by a deep convolutional neural network. Sci Rep. 2020;10:15245. doi: [10.1038/s41598-020-72270-6](https://doi.org/10.1038/s41598-020-72270-6).

4. Wu J-P, Ding W-Z, Wang Y-L, Liu S, Zhang X-Q, Yang Q, et al. Radiomics analysis of ultrasound to predict recurrence of hepatocellular carcinoma after microwave ablation. Int J Hyperthermia. 2022;39:595-604. doi: [10.1080/02656736.2022.2062463](https://doi.org/10.1080/02656736.2022.2062463).

5. Zhou L-Q, Wu X-L, Huang S-Y, Wu G-G, Ye H-R, Wei Q, et al. Lymph node metastasis prediction from primary breast cancer US images using deep learning. Radiology. 2020;294:19-28. doi: [10.1148/radiol.2019190372](https://doi.org/10.1148/radiol.2019190372).

6. Hu P, Gao Y, Zhang Y, Sun K. Ultrasound image-based deep learning to differentiate tubal-ovarian abscess from ovarian endometriosis cyst. Front Physiol. 2023;14:1101810. doi: [10.3389/fphys.2023.1101810](https://doi.org/10.3389/fphys.2023.1101810).
