# Supplementary material for: Multimodal model integrating ultrasound and demographic data for the diagnosis of knee osteoarthritis
Source: BMC Med Imaging. 2026 Apr 2;26:248. doi: 10.1186/s12880-026-02249-8 (PMC13169557; doi:10.1186/s12880-026-02249-8)
Supplement: Supplementary file 3 — Supplementary Material 3: File name: Additional file 3 Table S2. File format: .docx. Title of data: SHAP values for age, sex, and BMI for each CNN model. Description of data: This table lists the SHAP values for age, sex, and BMI across all CNN models, illustrating the contribution of demographic variables to the prediction output [file 12880_2026_2249_MOESM3_ESM.docx]

**Additional file Table S2.** SHAP values for age, sex, and BMI for each CNN model

| Network model | Category | Average SHAP value |
| --- | --- | --- |
| DenseNet169 | Age | 0.000079 |
|  | Sex | 0.000015 |
|  | BMI | -0.00046 |
| DenseNet201 | Age | -0.00004 |
|  | Sex | -0.000058 |
|  | BMI | 0.002366 |
| InceptionV3 | Age | 0.000061 |
|  | Sex | 0.000003 |
|  | BMI | 0.000151 |
| Inception-ResNet-v2 | Age | 0.000174 |
|  | Sex | -0.000022 |
|  | BMI | 0.000199 |
| ResNet50 | Age | -0.000004 |
|  | Sex | -0.000000006 |
|  | BMI | -0.0000008 |
| ResNet101 | Age | -0.000266 |
|  | Sex | 0.000048 |
|  | BMI | -0.000603 |
| ResNet152 | Age | 0.000519 |
|  | Sex | -0.000029 |
|  | BMI | 0.00067 |
| Xception | Age | 0.000005 |
|  | Sex | -0.000019 |
|  | BMI | -0.000492 |
| VGG16 | Age | 0.005326 |
|  | Sex | -0.000862 |
|  | BMI | -0.01367 |
| GoogLeNet | Age | -0.000889 |
|  | Sex | -0.000077 |
|  | BMI | 0.00034 |
| AlexNet | Age | 0.001419 |
|  | Sex | -0.000003 |
|  | BMI | -0.000438 |

BMI, body mass index; CNN, convolutional neural network; SHAP, SHapley Additive exPlanations
