# Supplementary material for: Multimodal model integrating ultrasound and demographic data for the diagnosis of knee osteoarthritis
Source: BMC Med Imaging. 2026 Apr 2;26:248. doi: 10.1186/s12880-026-02249-8 (PMC13169557; doi:10.1186/s12880-026-02249-8)
Supplement: Supplementary file 4 — Supplementary Material 4: File name: Additional file 4 Table S3. File format: .docx. Title of data: SHAP values for BMI for each CNN model. Description of data: This table summarizes the SHAP values representing the contribution of BMI to the prediction output for each CNN model [file 12880_2026_2249_MOESM4_ESM.docx]

**Additional file Table S3**. SHAP values for BMI for each CNN model

| Network mode | Average SHAP value |
| --- | --- |
| DenseNet169 | -0.000189 |
| DenseNet201 | -0.000157 |
| InceptionV3 | -0.000415 |
| Inception-ResNet-v2 | 0.000055 |
| ResNet50 | -0.000258 |
| ResNet101 | 0.000138 |
| ResNet152 | -0.000002 |
| Xception | -0.00075 |
| VGG16 | 0.000075 |
| GoogLeNet | 0.000087 |
| AlexNet | 0.000341 |

BMI, body mass index; CNN, convolutional neural network; SHAP, SHapley Additive exPlanations
