# Supplementary material for: Multimodal model integrating ultrasound and demographic data for the diagnosis of knee osteoarthritis
Source: BMC Med Imaging. 2026 Apr 2;26:248. doi: 10.1186/s12880-026-02249-8 (PMC13169557; doi:10.1186/s12880-026-02249-8)
Supplement: Supplementary file 5 — Supplementary Material 5: File name: Additional file 5 Table S4. File format: .docx. Title of data: Results of previous studies on multiclass classification. Description of data: This table provides a summary of previous studies presenting multiclass classification for knee osteoarthritis, including target classes, methods, and performance metrics [file 12880_2026_2249_MOESM5_ESM.docx]

**Additional file Table S4.** Results of previous studies on multi-class classification

| Author | Modality | Inspection method | Evaluation target | Classification type | Classification content | Sensitivity (%) | Specificity (%) | PPV (%) | NPV (%) | F1 score (%) | Diagnostic accuracy (％） |
| --- | --- | --- | --- | --- | --- | --- | --- | --- | --- | --- | --- |
| Mohammed et al. [1] | X-ray | ResNet-101 | Image alone | 5 classes | KL0 | 64.0 | 83.3 | 59.3 | 85.9 | 61.6 | 69.0 |
|  |  |  |  |  | KL1 | 48.2 | 85.6 | 55.5 | 81.6 | 51.6 |  |
|  |  |  |  |  | KL2 | 59.7 | 82.3 | 57.2 | 83.7 | 58.4 |  |
|  |  |  |  |  | KL3 | 57.1 | 91.0 | 61.8 | 89.3 | 59.3 |  |
|  |  |  |  |  | KL4 | 75.2 | 93.9 | 67.5 | 95.7 | 71.1 |  |
|  |  |  |  | 3 classes | Mild OA: KL2 | 92.8 | 85.8 | 91.4 | 88.0 | 92.1 | 89.0 |
|  |  |  |  |  | Moderate OA: KL3 | 81.2 | 92.6 | 83.0 | 91.6 | 82.1 |  |
|  |  |  |  |  | Severe OA: KL4 | 88.2 | 99.4 | 91.8 | 99.1 | 99.0 |  |
| Pi et al. [2] | X-ray | Ensemble using multiple CNNs such as ResNet101, EfficientNet, DenseNet, etc. | Image alone | 5 classes | KL0 | 89.8 | 95.0 | 79.2 | 97.9 | 84.2 | 76.9 |
|  |  |  |  |  | KL1 | 39.5 | 90.4 | 53.2 | 84.6 | 45.4 |  |
|  |  |  |  |  | KL2 | 79.2 | 88.8 | 76.8 | 91.1 | 78.0 |  |
|  |  |  |  |  | KL3 | 83.4 | 97.1 | 91.2 | 94.9 | 87.1 |  |
|  |  |  |  |  | KL4 | 84.3 | 99.6 | 93.5 | 97.9 | 88.7 |  |
| Kim et al. [3] | X-rays | SE-ResNet (ResNet with Squeeze-and-Excitation mechanism) | Image alone | 5 classes | KL0 | 64.9 | 94.8 | 67.6 | 94.2 | 66.2 | 51.9 |
|  |  |  |  |  | KL1 | 94.0 | 49.6 | 35.4 | 96.6 | 51.2 |  |
|  |  |  |  |  | KL2 | 80.0 | 51.2 | 34.4 | 88.9 | 48.0 |  |
|  |  |  |  |  | KL3 | 75.5 | 78.3 | 58.1 | 88.9 | 65.6 |  |
|  |  |  |  |  | KL4 | 77.4 | 90.7 | 48.8 | 97.2 | 59.7 |  |
|  |  |  | Image + Background Factor |  | KL0 | 97.3 | 92.3 | 67.9 | 99.5 | 79.8 | 61.6 |
|  |  |  |  |  | KL1 | 92.3 | 56.9 | 38.6 | 96.2 | 54.1 |  |
|  |  |  |  |  | KL2 | 76.0 | 61.6 | 38.8 | 88.9 | 50.4 |  |
|  |  |  |  |  | KL3 | 73.5 | 81.8 | 61.7 | 88.6 | 67.1 |  |
|  |  |  |  |  | KL4 | 73.6 | 91.4 | 49.4 | 96.8 | 59.1 |  |
| Tiulpin and Saarakkala [4] | X-ray | SE-ResNet-50 + SE-ResNeXt50-32x4d (ensemble) | Image alone | 5 classes | KL0 | 63.0 | 97.9 | 88.3 | 91.4 | 73.5 | 66.7 |
|  |  |  |  |  | KL1 | 11.0 | 96.6 | 44.9 | 81.3 | 17.7 |  |
|  |  |  |  |  | KL2 | 79.8 | 73.6 | 43.0 | 93.6 | 55.9 |  |
|  |  |  |  |  | KL3 | 84.8 | 93.0 | 75.0 | 96.1 | 79.6 |  |
|  |  |  |  |  | KL4 | 94.9 | 97.3 | 89.7 | 98.7 | 92.2 |  |
| Antony et al. [5] | X-rays | Homemade lightweight CNN (classification + regression) | Image alone | 5 classes | KL0 | 80.0 | 80.0 | 68.0 | 80.0 | 74.0 | 63.4 |
|  |  |  |  |  | KL1 | 15.0 | 15.0 | 32.0 | 15.0 | 20.0 |  |
|  |  |  |  |  | KL2 | 63.0 | 63.0 | 53.0 | 63.0 | 58.0 |  |
|  |  |  |  |  | KL3 | 74.0 | 74.0 | 78.0 | 74.0 | 76.0 |  |
|  |  |  |  |  | KL4 | 75.0 | 75.0 | 81.0 | 75.0 | 78.0 |  |
| Guida et al. [6] | MRI | Proprietary architecture using | Image alone | 5 classes | KL0 | 65.0 | 81.3 | 46.4 | 90.3 | 54.3 | 54.0 |
|  |  |  |  |  | KL1 | 7.5 | 97.5 | 42.9 | 80.8 | 12.9 |  |
|  |  |  |  |  | KL2 | 65.0 | 77.5 | 41.9 | 99.0 | 51.1 |  |
|  |  |  |  |  | KL3 | 46.2 | 95.0 | 69.2 | 87.9 | 55.4 |  |
|  |  |  |  |  | KL4 | 95.0 | 93.8 | 79.2 | 98.7 | 86.2 |  |

CNN, convolutional neural network; KL, Kellgren–Lawrence; OA, osteoarthritis; MRI, magnetic resonance imaging; NPV, negative predictive value; PPV, positive predictive value

**Additional reference**

1. Mohammed AS, Hasanaath AA, Latif G, Bashar A. Knee osteoarthritis detection and severity classification using residual neural networks on preprocessed X-ray images. Diagnostics (Basel). 2023;13:1380. doi: [10.3390/diagnostics13081380](https://doi.org/10.3390/diagnostics13081380).

2. Pi S-W, Lee B-D, Lee MS, Lee HJ. Ensemble deep-learning networks for automated osteoarthritis grading in knee X-ray images. Sci Rep. 2023;13:22887. doi: [10.1038/s41598-023-50210-4](https://doi.org/10.1038/s41598-023-50210-4).

3. Kim DH, Lee KJ, Choi D, Lee JI, Choi HG, Lee YS. Can additional patient information improve the diagnostic performance of deep learning for the interpretation of knee osteoarthritis severity. J Clin Med. 2020;9:3341. doi: [10.3390/jcm9103341](https://doi.org/10.3390/jcm9103341).

4. Tiulpin A, Saarakkala S. Automatic grading of individual knee osteoarthritis features in plain radiographs using deep convolutional neural networks. Diagnostics (Basel). 2020;10:932. doi: [10.3390/diagnostics10110932](https://doi.org/10.3390/diagnostics10110932).

5. Antony J, McGuinness K, O’Connor NE, Moran K. Automatic detection of knee joints and quantification of knee osteoarthritis severity using convolutional neural networks. In: IEEE International Symposium on Biomedical Imaging (ISBI); 2017. p. 1203-6.

6. Guida C, Zhang M, Shan J. Knee osteoarthritis classification using 3D CNN and MRI. Appl Sci. 2021;11:5196. doi: [10.3390/app11115196](https://doi.org/10.3390/app11115196).
