# Supplementary material for: Multimodal model integrating ultrasound and demographic data for the diagnosis of knee osteoarthritis
Source: BMC Med Imaging. 2026 Apr 2;26:248. doi: 10.1186/s12880-026-02249-8 (PMC13169557; doi:10.1186/s12880-026-02249-8)
Supplement: Supplementary file 6 — Supplementary Material 6: File name: Additional file 6 Table S5. File format: .docx. Title of data: Comparison of the diagnostic accuracies of the two classifications. Description of data: This table compares diagnostic accuracy of two classification strategies (KL 0–1 vs. 2–4 and KL 0 vs. 1–4), highlighting differences in sensitivity, specificity, and overall performance [file 12880_2026_2249_MOESM6_ESM.docx]

**Additional file Table S5.** Comparison of the diagnostic accuracies of the two classifications

| Author | Modality | Assessment method | Testing method | Assessment method | Classification type | Classification details | AUC | Sensitivity (%) | Specificity (%) | PPV (%) | NPV (%) | F1 score (%) | Diagnostic accuracy (%) | Note |
| --- | --- | --- | --- | --- | --- | --- | --- | --- | --- | --- | --- | --- | --- | --- |
| Results of own study | US | CNN | ResNet50 | Image alone | 2 classes | OA vs. non-OA: KL 0–1 vs. KL 2–4 | 0.82 | 80.2 | 78.8 | 87.5 | 68.3 | 83.7 | 80.0 |  |
|  |  |  | VGG16 |  |  | Severity Classification: KL 2 vs. KL 3–4 | 0.88 | 94.3 | 77.0 | 70.2 | 95.9 | 82.2 | 83.3 |  |
|  |  |  | InceptionV3 | Image + background factor | 2 classes | OA vs. non-OA: KL 0–1 vs. KL 2–4 | 0.89 | 80.2 | 86.5 | 91.6 | 70.3 | 86.0 | 82.4 |  |
|  |  |  | ResNet50 |  |  | Severity Classification: KL 2 vs KL 3–4 | 0.90 | 91.4 | 75.4 | 70.8 | 100.0 | 82.9 | 81.3 |  |
| Kiso et al. [1] | US | Radiomics | Statistical model with single feature | Image alone | 2 classes | OA vs. non-OA: KL 0–1 vs. KL 2–4 | NA | 47.0 | 98.0 | 97.0 | 52.0 | NA | 65.8 |  |
|  |  |  |  |  |  | Severity Classification: KL 2 vs. KL 3–4 | NA | 78.0 | 86.0 | 72.0 | 90.0 | NA | 83.6 |  |
|  |  |  | Machine Learning Model with Multiple Features | Image alone | 2 classes | OA vs. non-OA: KL 0–1 vs. KL 2–4 | 0.79 | 71.0 | 82.0 | 88.0 | 59.0 | 79.0 | 75.0 | * |
|  |  |  |  |  |  | Severity classification: KL 2 vs. KL 3–4 | 0.92 | 81.0 | 85.0 | 76.0 | 88.0 | 78.0 | 83.0 |  |
|  |  |  |  | Image + Background Factor | 2 classes | OA vs. non-OA: KL 0–1 vs. KL 2–4 | 0.87 | 81.0 | 80.0 | 89.0 | 69.0 | 85.0 | 81.0 |  |
|  |  |  |  |  |  | Severity classification: KL 2 vs. KL 3–4 | 0.92 | 78.0 | 85.0 | 76.0 | 86.0 | 77.0 | 82.0 |  |
| Kiso et al. [2] | US | Measurements | TOH-DBB Index | Image alone | 2 classes | OA vs. non-OA: KL 0–1 vs. KL 2–4 | NA | 80.4 | 75.9 | 85.4 | 68.8 | NA | 78.7 |  |
|  |  |  |  |  |  | Severity Classification: KL 2 vs. KL 3–4 | NA | 71.4 | 85.2 | 71.4 | 85.2 | NA | 80.5 |  |
| Mohammed et al. [3] | X-ray | CNN | ResNet-101 | Image alone | 2 classes | OA vs. non-OA: KL 0–1 vs. KL 2–4 | NA | 94.0 | 79.0 | 85.0 | 91.0 | 89.0 | 87.5 |  |
| Tiulpin et al. [4] | X-ray | CNN | ResNet-34 | Image alone | 2 classes | OA vs. non-OA: KL 0–1 vs. KL 2–4 | 0.93 | 80.0 | 91.0 | 87.0 | 86.0 | 83.0 | 86.0 | † |
| Yeoh et al. [5] | MRI | CNN | ResNet34 | Image alone | 2 classes | OA vs. non-OA: KL 0–1 vs. KL 2–4 | 0.93 | 88.0 | 88.0 | 87.0 | NA | 87.0 | 87.5 | ‡ |
|  |  |  | ResNet18 |  |  |  | 0.95 | 100 | 63.0 | 99.0 | NA | 82.9 | 62.5 |  |
|  |  |  | VGG19 |  |  |  | 0.91 | 100 | 68.8 | 91.4 | NA | 86.0 | 68.8 |  |
| Guida et al. [6] | MRI | CNN | 3D CNN | Image alone | 2 classes | OA vs. non-OA: KL 0–1 vs. KL 2–4 | 0.91 | 81.7 | 84.5 | 84.5 | 83.1 | 83.1 | 83.1 | § |
| Pedoia et al. [7] | MRI | CNN | DenseNet | Image alone | 2 classes | OA vs. non-OA: KL 0–1 vs. KL 2–4 | NA | 74.5 | 76.1 | NA | NA | NA | NA |  |
|  |  |  |  | Image + Background Factor | 2 classes | OA vs. non-OA: KL 0–1 vs. KL 2–4 | 0.83 | 77.0 | 77.9 | NA | NA | NA | NA |  |

* Based on the results of Kiso et al., we selected the models (random forest for images alone and logistic regression for images with background factors) that achieved the best balance of sensitivity, AUC, and F-score and minimized the risk of missing OA cases. The model (discriminant analyzer for both image alone and image plus background factors) with the highest sensitivity and NPV and the lowest risk of missing severe cases was selected to determine severity.

† Based on the confusion matrix in Figure 4a by Tiulpin et al., the main diagnostic metrics (sensitivity, specificity, PPV, NPV, F1 score, and diagnostic accuracy) were independently calculated by the author based on the revised KL grades. Grades 2 and above denoted OA, whereas grades 1 and below denoted non-OA.

‡ Diagnostic accuracy, according to Yeoh et al., is calculated as the average of sensitivity and specificity.

§ The PPVs and NPVs were independently recalculated by the author based on the reported sensitivities, specificities, and class distributions (KL grades 0–1 and 2–4 for 220 cases each) in the test set by Guida et al. The calculations are based on the assumption of class balance rather than the values explicitly stated in the original study.

AUC, area under the curve; KL, Kellgren–Lawrence; OA, osteoarthritis; CNN, convolutional neural network; MRI, magnetic resonance imaging; NPV, negative predictive value; PPV, positive predictive value; US, ultrasonography

**Additional reference**

1. Kiso T, Okada Y, Kawata S, Shichiji K, Okumura E, Hatsumi N, et al. Ultrasound-based radiomics and machine learning for enhanced diagnosis of knee osteoarthritis: evaluation of diagnostic accuracy, sensitivity, specificity, and predictive value. Eur J Radiol Open. 2025;14:100649. doi: [10.1016/j.ejro.2025.100649](https://doi.org/10.1016/j.ejro.2025.100649).

2. Kiso T, Okada Y, Kawata S, Shichiji K, Okumura E, Hatsumi N, et al. Diagnostic accuracy of a novel ultrasound imaging index for knee osteoarthritis: evaluation of sensitivity, specificity, and predictive values. J Clin Ultrasound. 2024;52:687-99. doi: [10.1002/jcu.23691](https://doi.org/10.1002/jcu.23691).

3. Mohammed AS, Hasanaath AA, Latif G, Bashar A. Knee osteoarthritis detection and severity classification using residual neural networks on preprocessed X-ray images. Diagnostics (Basel). 2023;13:1380. doi: [10.3390/diagnostics13081380](https://doi.org/10.3390/diagnostics13081380).

4. Tiulpin A, Thevenot J, Rahtu E, Lehenkari P, Saarakkala S. Automatic knee osteoarthritis diagnosis from plain radiographs: A deep learning-based approach. Sci Rep. 2018;8:1727. doi: [10.1038/s41598-018-20132-7](https://doi.org/10.1038/s41598-018-20132-7).

5. Yeoh PSQ, Lai KW, Goh SL, Hasikin K, Wu X, Li P. Transfer learning-assisted 3D deep learning models for knee osteoarthritis detection: data from the osteoarthritis initiative. Front Bioeng Biotechnol. 2023;11:1164655. doi: [10.3389/fbioe.2023.1164655](https://doi.org/10.3389/fbioe.2023.1164655).

6. Guida C, Zhang M, Shan J. Knee osteoarthritis classification using 3D CNN and MRI. Appl Sci. 2021;11:5196. doi: [10.3390/app11115196](https://doi.org/10.3390/app11115196).

7. Pedoia V, Lee J, Norman B, Link TM, Majumdar S. Diagnosing osteoarthritis from T2 maps using deep learning: an analysis of the entire osteoarthritis Initiative baseline cohort. Osteoarthr Cartil. 2019;27:1002-10. doi: [10.1016/j.joca.2019.02.800](https://doi.org/10.1016/j.joca.2019.02.800).
